# Model-Based Analysis for Qualitative Data: An Application in *Drosophila* Germline Stem Cell Regulation

**DOI:** 10.1371/journal.pcbi.1003498

**Published:** 2014-03-13

**Authors:** Michael Pargett, Ann E. Rundell, Gregery T. Buzzard, David M. Umulis

**Affiliations:** 1 Weldon School of Biomedical Engineering, Purdue University, West Lafayette, Indiana, United States of America; 2 Department of Mathematics, Purdue University, West Lafayette, Indiana, United States of America; 3 Agricultural and Biological Engineering, Purdue University, West Lafayette, Indiana, United States of America; University of Virginia, United States of America

## Abstract

Discovery in developmental biology is often driven by intuition that relies on the integration of multiple types of data such as fluorescent images, phenotypes, and the outcomes of biochemical assays. Mathematical modeling helps elucidate the biological mechanisms at play as the networks become increasingly large and complex. However, the available data is frequently under-utilized due to incompatibility with quantitative model tuning techniques. This is the case for stem cell regulation mechanisms explored in the *Drosophila* germarium through fluorescent immunohistochemistry. To enable better integration of biological data with modeling in this and similar situations, we have developed a general parameter estimation process to quantitatively optimize models with qualitative data. The process employs a modified version of the Optimal Scaling method from social and behavioral sciences, and multi-objective optimization to evaluate the trade-off between fitting different datasets (e.g. wild type vs. mutant). Using only published imaging data in the germarium, we first evaluated support for a published intracellular regulatory network by considering alternative connections of the same regulatory players. Simply screening networks against wild type data identified hundreds of feasible alternatives. Of these, five parsimonious variants were found and compared by multi-objective analysis including mutant data and dynamic constraints. With these data, the current model is supported over the alternatives, but support for a biochemically observed feedback element is weak (i.e. these data do not measure the feedback effect well). When also comparing new hypothetical models, the available data do not discriminate. To begin addressing the limitations in data, we performed a model-based experiment design and provide recommendations for experiments to refine model parameters and discriminate increasingly complex hypotheses.

## Author Summary

We developed a process to quantitatively fit mathematical models using qualitative data, and applied it in the study of how stem cells are regulated in the fruit fly ovary. The available published data we collected are fluorescent images of protein and mRNA expression from genetic experiments. Despite lacking quantitative data, the new process makes available quantitative model analysis techniques to reliably compare different models and guide future experiments. We found that the current consensus regulatory model is supported, but that the data are indeed insufficient to address more complex hypotheses. With the quantitatively fit models, we evaluated hypothetical experiments and estimated which future measurements should best refine or test models. The model fitting process we have developed is applicable to many biological studies where qualitative data are common, and can accelerate progress through more efficient experimentation.

## Introduction

Biological systems are often characterized using qualitative data, such as stained images, immunoblots, microarrays, or observations of cell morphology, rather than absolute values (e.g. molecular concentration). Such qualitative data typically show relative relationships in how a system characteristic (e.g. expression of proteins or mRNA, morphology, phenotype) is distributed spatially, and/or changes with time or with genetic perturbations. These data are prevalent due to the complexity of biological systems and measurements, from spatial organization and dynamic behavior, to the need for multi-step reactions to generate a measurable signal, along with the wide variability of experimental factors (e.g. reagent concentrations, background interference, antibody quality and specificity) [1], [2]. In many cases, more time-consuming quantifiable measurements are sacrificed for improved throughput and spatial resolution [3], though the resulting uncertainty in absolute value, range and resolution is limiting, particularly as applied in mathematical models. In this study, we address these limitations in model-data integration in the context of a stem cell niche in the *Drosophila* germarium, as the available data are largely qualitative and it has become a model system from which we hope to gain insight into stem cell regulation.

**Figure 1:**
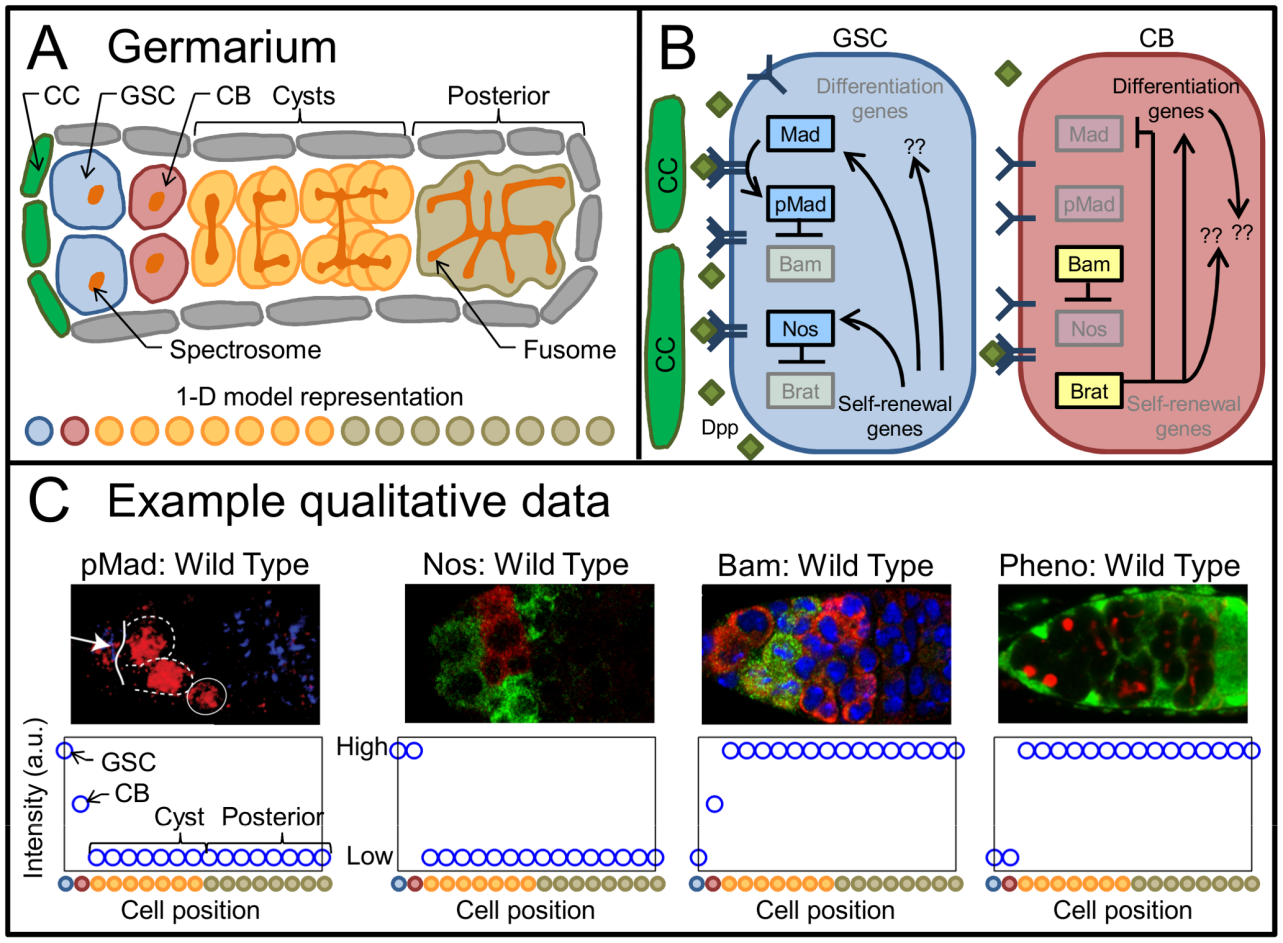
*Drosophila* germarium system and data. A) Diagram of Wild Type germarium structure with anterior to the left, showing cap cells (CC), germline stem cells (GSC), cystoblasts (CB), and cysts. Below, a color-matched schematic of the 1 dimensional model used in this study. B) Diagram of signaling in the anterior germarium, showing the internal regulatory state of the GSC (left), and CB (right). Yellow and blue boxes refer to differentiation- and self-renewal-promoting elements, respectively. C) Examples of typical images (upper) and qualitative interpretations (lower) comprising available data. Qualitative interpretations are mappings of relative intensity (fluorescent, colorimetric, etc.) and original author interpretations to the 1-D model, indicated at bottom. Relevant color channels per image are (from left to right): pMad in red, Nos in red, Bam in green, Phenotype showing spectrosomes in red. Images reproduced/adapted from [57] (1st from left), [58] (2nd and 3rd from left), and [59] (right).

Illustrated in Figure 1A, each oblong germarium houses 2–3 germline stem cells (GSC) associated with the cap cells (CC) at the anterior end. In the course of differentiating, GSC progeny transition through a cystoblast phase (CB, single cells beginning to express differentiation factors), then divide repeatedly forming cysts interconnected by a fusome. In undifferentiated cells, the fusome structure is isolated and spherical, referred to as a spectrosome (fusome/spectrosome morphology is a common observation). The regulation of stem cell self-renewal vs. differentiation depends on signaling by Decapentaplegic (Dpp), a bone morphogenic protein (BMP) ligand homologue, which is expressed by the cap cells. As illustrated in Figure 1B, the GSC is maintained by Dpp signaling, mediated through surface receptors that promote phosphorylation of Mad to pMad. pMad acts as an input to a regulatory network, including (at least) Bam, Nos and Brat. For more complete coverage of germarium structure and function, we recommend recent reviews [4]–[6].

Multiple types of data inform GSC regulation in the germarium. Most prominently, spatial distribution data are published in the form of fluorescent intensity for several proteins (in some cases mRNA) in wild type and in different mutant backgrounds (examples shown in Figure 1C). Were all of these data collected via fully quantitative techniques, they would be approachable with common model fitting techniques (e.g. least squared error regression). However, measuring precise quantitative levels of protein or mRNA in vivo continues to be very technically challenging, and the existing qualitative data are not directly comparable for optimizing models by typical regression. The scaling of fluorescent intensity differs for each molecular species, relying on different antibodies and the reaction conditions for each sample, and in some instances data are aggregated from different publications. Individually, these qualitative data provide loose constraints and to be effective they must be considered simultaneously, for which new methods are needed. Furthermore, the data are provided by three principle types of observations: wild type protein distributions at a single time point, distributions for different subsets of the components in mutant and/or ectopic expression experiments, and estimates of the time between cell cycles that provide a dynamic constraint. Lacking further information, it is unclear if one of these disparate observation types is better for a model to satisfy than another, which leaves only limited meaning to a single best parameter set.

To assess feasible mechanisms in this system (and others with qualitative constraints), we developed an integrated strategy comprising two applications seldom used with biological models: Optimal Scaling to quantitatively estimate model fitness, and Pareto multi-objective optimization to simultaneously consider multiple disparate types of data. Though we apply it here in a study of stem cell regulation in the *Drosophila* germarium, the procedure is applicable to any quantitative model.

This study integrates research in three primary areas: (1) optimization with qualitative data and the Optimal Scaling procedure, (2) the problem of using multiple disparate datasets, and multi-objective optimization as a robust solution, and (3) modeling of stem cell regulation in the *Drosophila* germarium. The remainder of this introduction is dedicated to informing these three areas.

### Optimization and qualitative data: Optimal Scaling

**Figure 2:**
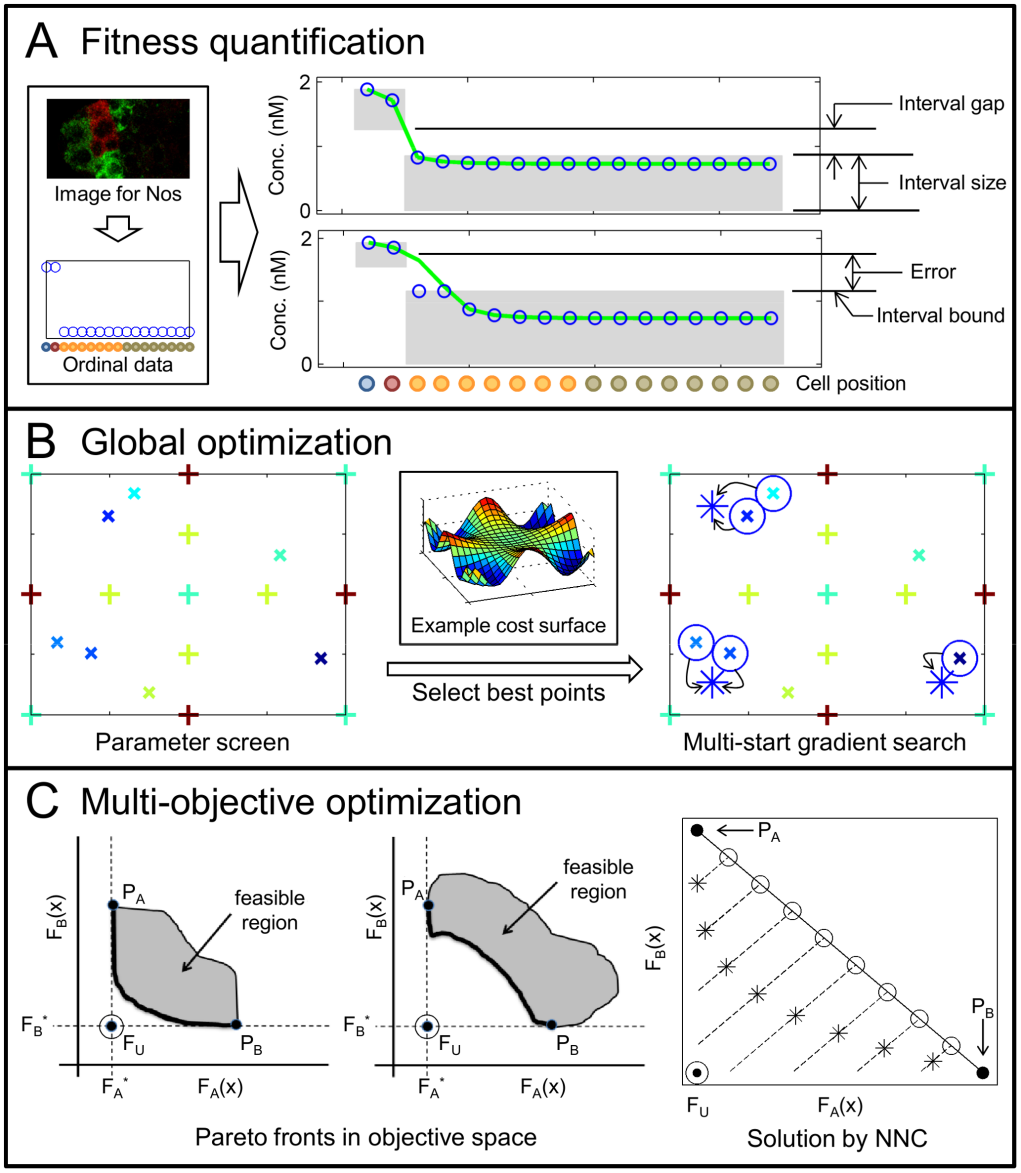
Estimation of representative parameters. A) Diagram of fitness quantification by Optimal Scaling. Images are mapped to qualitative data (left, image reproduced/adapted from [58]). Surrogate data (blue 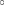) are chosen to best fit model outputs (green line) within intervals that match data (shaded boxes). Intervals are additionally constrained by minimum gaps and sizes. B) Diagram of the global optimization procedure. (Left) Parameter space is screened by sparse grid (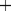) and pseudo-random sampling (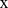)A. Color indicates cost (blue: low, red: high). (Right) Multiple gradient-based searches are started from the best samples (blue 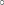), and find local minima (blue 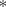). Finding all minima is not guaranteed (no solution in upper right quadrant). C) Diagram of the multiobjective optimization procedure. (Left and center) Example Pareto fronts (solid line) for objectives 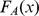 and 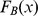, showing Anchor points (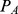 and 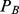), and Utopian points (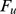). (Right) Example solution via the modified NNC method, showing: Anchor points (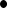), Utopian (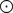), gradient search starts (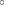), normal constraints (dashed line), gradient solutions (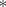).

Optimization algorithms attempt to find a parameter set (or point, i.e. a value for each uncertain parameter) that gives the best value for some objective defining model fitness, typically the error between model predictions and data; they are commonly identified as either local or global methods (Figure 2B illustrates these as applied in this study). Local optimization starts at a specific parameter set and selects a search direction and step based on the gradient, i.e. how much the error changes with small parameter changes. Global methods use the fitness evaluated for a sampling of parameter sets to then select new samples expected to improve (algorithmic details vary). Qualitative data, such as the fluorescent images of the germarium, define predominantly binary fitness criteria; either the model outputs satisfy the observation or not. They provide no gradient information and discontinuous changes in fitness that may be difficult to identify. Optimization procedures are likely to fail to see where a better solution might lie if a sample did not happen to be placed there. As a result, biological model parameters have typically been estimated either using only data that is quantitative, or by the modeler manually adjusting parameters based on intuition, a very time-consuming process.

To design a general procedure for optimization to qualitative data, we considered past efforts in several fields that have addressed aspects of the problem. We predominantly build on the Optimal Scaling method, reviewed below, but it is informative to comment on alternative techniques available. In statistics, regression to qualitative data has a long history [7], but in contrast with the mechanistic biological context, only minimal models are used. These statistical models are typically linear with some assumed structure on the data (i.e. a function such as logit or probit is applied to the model values). Thresholds are defined to subdivide the continuous model output into intervals, and map each interval to a discrete qualitative output (e.g. high and low, or a phenotype name). The reliance on model linearity limits the immediate utility of past statistical approaches for the non-linear models at hand. In complex model analysis, behavior discrimination [8] has recently been described to define thresholds among different model behaviors, but could be applied to model tuning with qualitative data. It relies on mathematical descriptions of each qualitative behavior to create quantitative metrics to evaluate how near a model is to satisfying each behavior. Defined behaviors can range from simple thresholds to complex time-dependent relationships. A conceptual compromise, Optimal Scaling [9] is an older approach that originated in the social sciences. Similar to behavior discrimination, it evaluates a distance from the point of satisfaction, but is more directly oriented toward model tuning. It also resembles statistical regression problems, but while its past use has been with simple models, it is more generally applicable to complex cases (i.e. non-linear models). For a particular model output, Optimal Scaling uses regression to estimate the optimal quantitative values likely to have generated the qualitative data, i.e. the best-case fit to that model output. While each of these approaches estimates a quantitative fitness, Optimal Scaling offers particularly broad applicability and a focus on the feasible values of the real system.

The Optimal Scaling process is illustrated in Figure 2A, and details are provided in Methods. Each time a model output is considered, Optimal Scaling defines quantitative values to replace the qualitative observations; we refer to these as surrogate data (illustrated as blue circles in Figure 2A, right). The surrogate data are intended to represent what could have existed in the true system. To evaluate the best-case fitness to the given model output, the surrogate data values are optimized within the constraint that they still satisfy the qualitative observations (constraints shown as shaded boxes in Figure 2A). The quantitative error between these optimal values and the model output then defines the model fitness, and may be used as the objective for existing optimization techniques. As originally presented for regression of simple models in the social sciences, Optimal Scaling is alternated with a least squares optimization of parameter values [9], [10]. However, for more complex models, the necessary convexity of that optimization scheme can not be guaranteed. Instead, to apply global and multi-objective optimization techniques, we nest the optimal scaling step fully within the parameter estimation problem [1] (i.e. optimal scaling is performed explicitly for every parameter set evaluated). For details on the optimization process, see Methods.

### Using disparate datasets: Multi-objective optimization

The Optimal Scaling procedure addresses model fitness to the qualitative distributions from germarium images (examples in Figure 1C), but the uncertainty among the different observation types remains. For quantitative data, the trade-off between satisfying each type would be informed by measured experimental variance. For these qualitative data, we suggest that the problem can be viewed as having multiple objectives, i.e. fitting each type of data as a separate objective (as described in general in [1]). In this way, the risk of bias in estimating a single best parameter set is mitigated and a more complete perspective on model performance constructed by evaluating the continuous trade-off among fitting the different data types. An approach that originated in economics and is commonly applied in design optimization, the multi-objective Pareto optimality concept focuses on determining a well-spaced set of points describing this trade-off, each of which corresponds to an optimal point for a different weighting among objectives [11]. Therein, a point is considered Pareto optimal if no other points improve one objective without compromising another.

Evaluating a set of Pareto optimal points (termed the Pareto front, demonstrated in Figure 2C) comes at a significant computational cost. It is useful to minimize the dimension of the multiobjective problem and group the most similar data together. While we consider the germarium data grouped by the type of observation, data can be grouped in a variety of other ways as suits the problem at hand, including the quality of data (e.g. nominal, ordinal, ratiometric, etc.), or the measurement technique used. The Pareto front is described by plotting the Pareto optimal points on the objective space (e.g. fitness to wild type data vs. to mutant data). Reflected in its placement and curvature, the Pareto front shows the trade-off between objectives, such as how much wild type fitness must be sacrificed to better fit mutants. Accordingly, we can then use the Pareto front to compare the performance of different models.

In this study we analyze simplified spatio-temporal models of the germarium subject to a compiled group of available qualitative data by estimating quantitative fitness through Optimal Scaling. To robustly capture data-consistent model behavior, we use multi-objective optimization to estimate a group of representative model parameter sets (Representatives). With this approach, we are able to refine predictive estimates of system behavior, discriminate among multiple models, and estimate the merit of future experiments. To develop and demonstrate the approach in the germarium, we compare alternative regulatory networks generated by a naive screen, as well as mechanistic hypotheses informed by current evidence, including a model based on previous work [12]. We then estimate Representative parameter sets (Pareto points, in this study) for each model and discriminate among models based on their simultaneous fitness to published qualitative protein and mRNA distribution data from wild type and mutant organisms. Using the Representatives for each model, we assess which data and parameters should be considered in expanding on the current models, and estimate which future experiments will be most informative by model-based experiment design.

## Results/Discussion

Stem cell regulation in the germarium has been represented by a variety of conceptual models [4]–[6] along with a spatio-temporal mathematical model [12]. Among these, the consensus regulatory network most widely supported is as shown in Figure 1B, referred to herein as the Core network. pMad, Bam, Nos, and Brat form a chain of repressors that results in bistable behavior with either pMad and Nos present (the self-renewing state, Figure 1B, left) or Bam and Brat present (the differentiating state, Figure 1B, right). This network is well supported through genetic and protein interaction studies, as well as modeling analysis [12], [13] and serves as the starting point for the mathematical modeling further developed herein. As indicated by question marks in Figure 1B, additional components and interactions are unknown, but expected. Examples include miRNA mediated repression [14], ligand endocytosis [15], [16], modification of the extracellular environment [17]–[20], and a variety of cell contact mediated mechanisms [21], [22].

### Compiled protein expression data

Data were compiled from published images of protein expression across wild type and mutant germaria. All of the data used (Table 1) are qualitative, giving relative expression of proteins, as shown in Figure 1. Phenotype data are common and indicate fusome morphology (e.g. Figure 1C, image on right, showing in red the spectrosomes as round and fusomes as branched). We correlate the fusome development to Brat expression as an indicator of differentiation. We consider the germarium divided into 4 regions: GSC, CB, Cyst and Posterior. The mapping of these regions onto our 1 dimensional models is shown in Figure 1A lower, indicated by color (See also Figure S1 in Supporting Information, Text S1, and refer to Methods for modeling). We provide this color map as a reference for model outputs throughout the analysis.

**Table 1:** Table of data employed to fit models.

Index	Experiment	Measurement	Reference
1	Wild Type	Nos	Casanueva, 2004 [25]
2	Wild Type	pMad	
3	Wild Type	Bam	
4	Wild Type	Phenotype	
5	Bam −/−	Phenotype	
6	Bam −/−	pMad	
7	Bam −/−	Nos	Li, 2009 [60]
8	dMyc OE	pMad	Rhiner, 2009 [16]
9	dMyc −/−	pMad	
10	dMyc +/−B	Phenotype	
11	dMyc −/−, dMyc OE	pMad	
12	Wild Type	Brat	Harris, 2011 [12]
13	Bam −/−	Brat	
14	Nos −/−	Brat	
15	Brat −/−	pMad	
16	Brat −/−	Bam	
17	Dpp −/−	Phenotype	Xie, 1998 [61]
18	Dpp +/−B	Phenotype	
19	Bam +/−B	Phenotype	Shen, 2009 [62]
20	Nos +/−B	Phenotype	Maines, 2007 [63]
21	Wild Type, Dynamic^B^	pMad	Morris, 2011 [51]
22	Wild Type, Dynamic^B^	Bam	
23	Wild Type, Dynamic^B^	Nos	
24	Wild Type, Dynamic^B^	Brat	
25	Wild Type, Dynamic^B^	Phenotype	

^*B*^Assigned to Behavioral data category.

Example qualitative interpretations of data are provided below each image in Figure 1C, with a reference to the 1-D model. We note that Bam is known to be repressed by RBP9, which is present in the posterior region of the germarium [23]. However, the regulation of RBP9 remains unknown. We neglect this posterior repression on Bam in the data, as it is outside the scope of the models we test. Examining the qualitative interpretations, it is apparent that each observation provides only loose constraints, emphasizing the importance of considering many such observations simultaneously.

To separate the different types of observations used, we divide data among three categories and independently evaluate model satisfaction of (1) Wild Type observations, (2) Mutant observations, and (3) Behavioral observations. The Behavioral category includes both dynamic constraints, specifying how quickly the cells must respond, and negative phenotypes observed in mutants, which reflect robustness to some perturbations (indicated in Table 1). These categories were chosen both for biological interest and to aggregate data expected to be similar. For example, Mutants commonly exhibit an all or nothing response over the entire germarium, while Wild Type responses are more graded.

### Representative parameter estimation procedure

We developed a new approach to search for Representatives that best satisfy qualitative data, which incorporates three elements: (1) the novel application of Optimal Scaling to quantitatively estimate model fitness, (2) global optimization to select a single best solution for each objective, and (3) multi-objective optimization to find a set of Representatives irrespective of weighting among objectives. Our implementation of these techniques is illustrated in Figure 2. For details on each of these processes, consult Methods.

The quantification of model fitness by Optimal Scaling in this study is represented in Figure 2A. The procedure generates surrogate data (blue circles) that are required to lie within intervals that ensure consistency with qualitative data (shaded boxes). Model error is then calculated as a relative sum of squared error between surrogates and the model output (green line). Note that error is only non-zero when surrogates cannot be perfectly aligned with the model output, as in cell positions 3 and 4 in Figure 2 A. The optimization problem in Optimal Scaling is to select the intervals and surrogates that minimize the model error for a given model output.

The global parameter estimation process is depicted in Figure 2B. In this study, we address non-linear spatio-temporal systems with a minimum of 10 states and 18 uncertain parameters. When estimating parameters, dense parameter screening is infeasible and gradient-based searches are not expected to reliably arrive at a global solution, but identify local optima instead. To proceed, we employ a hybrid semi-deterministic approach comprising a sparse global screen followed by a multi-start gradient search. It is important to keep in mind that for these models, available optimization techniques do not guarantee globally optimal or unique solutions (note the unidentified local minimum in Figure 2B right).

Finally, to generate the set of representative model parameters, we use multi-objective optimization to find points on the Pareto front, as illustrated in Figure 2C. Here, we determine the Pareto points (the Representatives) using the Normalized Normal Constraint (NNC) method [24] (Figure 2C, right), with modifications to suit the problem at hand and take advantage of global screening (see Methods for more details). This method performs multiple single-objective gradient searches, with each restricted to lie on a different line so that resulting points are well spaced (dashed lines in Figure 2C, right).

### Germarium Core network performance

**Figure 3:**
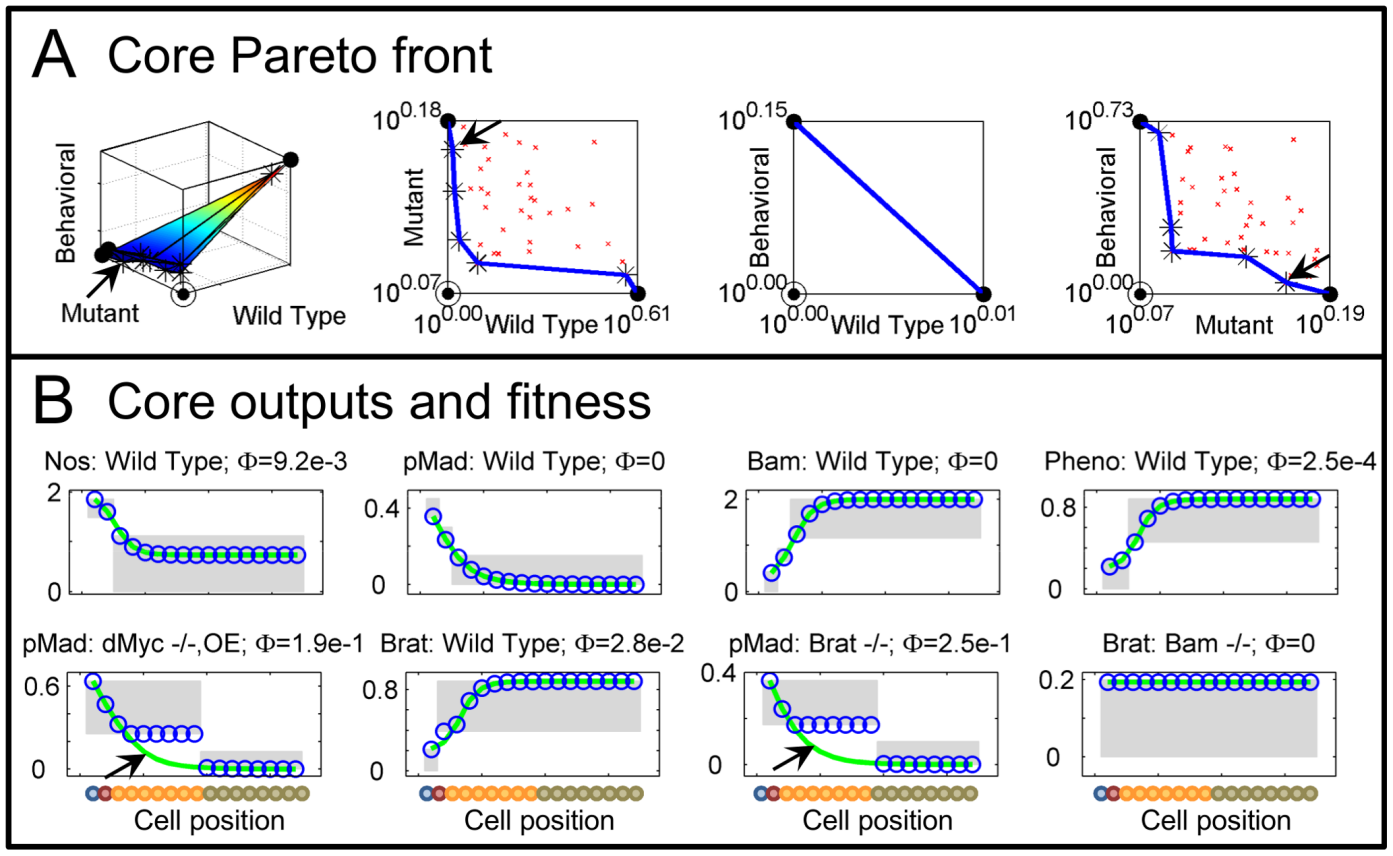
Parameter estimation in the Core network. A) Solved Pareto front for the Core network, showing the 3-D front (left) and projections (right 3 plots). Plotted in log space to accentuate small errors, showing: Pareto front (blue line), local solutions (red 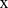), Pareto optimal solutions (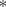), Anchor points (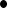), and Utopian point (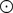). Projection views are bounded by the relevant Anchor points, so many solved points may not be visible and scales vary (e.g. Wild Type and Behavioral values are very similar and produce a negligible front with no Pareto points other than the Anchors, 3rd from left). B) Sample of model outputs and optimally scaled surrogate data for the Pareto point closest to the Utopian, indicated by black arrows in A. Plot titles indicate measurement: experiment; relative error (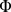). Notation as in Figure 2A.

The multi-objective approach reliably determines a set of Representatives for the germarium models. The Pareto front identified for the Core regulatory network (as depicted in Figure 1B) is shown in Figure 3.

The front is quite convex (toward the Utopian point), but with a significant trade-off between Wild Type and Mutant fitness (2nd from left). Behavioral fitness closely matches Wild Type (3rd from left, note the very small scale) and exhibits a similar trade-off with Mutant fitness (right). To illustrate fitness, Figure 3B presents examples of both well and poorly fit observations, for the Pareto point nearest the Utopian (arrows in Figure 3A), chosen by Euclidean distance to estimate a midpoint in the trade-off (fitness at nearby Pareto points was similar, data not shown). Most of the observations are satisfied, or nearly satisfied, at this point. The two largest misfits are pMad in a dMyc mutant with ectopic dMyc expression, and pMad in a Brat mutant (arrows in Figure 3B).

Examining the data and results for the Brat mutant leads to two important comments. First, we note that the interpretation of the Brat mutant phenotype may be overly aggressive (i.e. too many cells designated with high pMad), due to the discretization of the germarium into the 4 regions considered in this study. The Cyst region extends throughout the 2–8 cell cysts (cell 3–9 in the 1D model), but the indications from data of high pMad expression past the CB do not clearly extend throughout 8 cell cysts [12]. Second, while pMad signaling in the Brat mutant extends beyond the CB, that in Bam mutants does not [25], suggesting either an unknown regulatory interaction or inconsistency among experiments.

### Network inference supports the core structure

**Figure 4:**
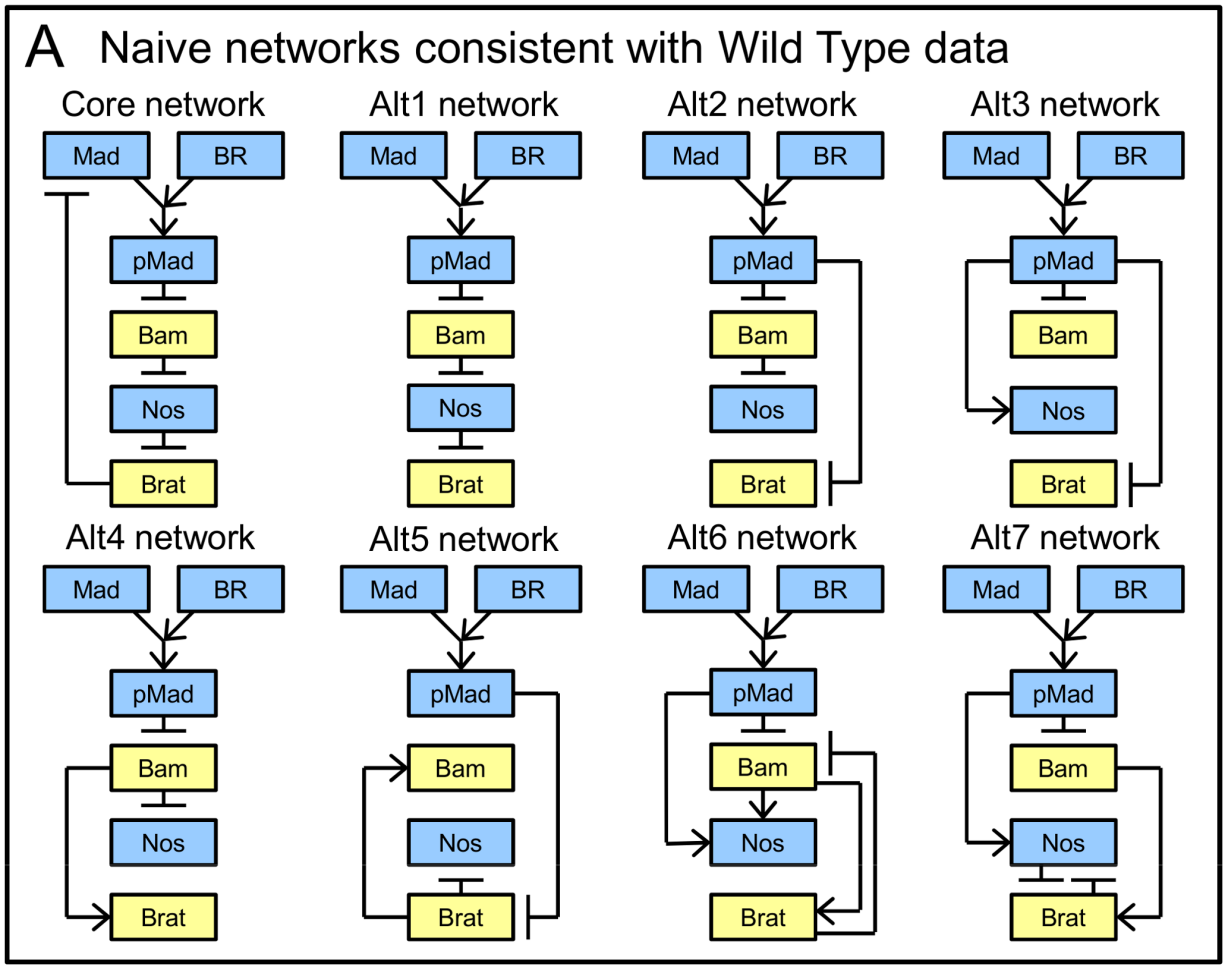
Naive regulatory networks. Yellow and blue boxes refer to differentiation- and self-renewal-promoting elements, respectively.

To evaluate the Core model in our framework, we compare alternative connections of its regulatory elements, pMad, Bam, Nos, and Brat (different model structures, i.e. rewiring of network edges). Through a simple network inference problem focused on Wild Type data only, we performed a broad screen of alternative networks and identified a set of feasible networks to more thoroughly evaluate, shown in Figure 4. Considering only Wild Type fitness, we tested the ∼65 k alternatives with only inhibitory connections and additionally performed searches for alternatives that include activation, beginning with 250 k samples. Refer to Methods and Supporting Information (Text S1) for details. Due to the sparse qualitative data, many networks (hundreds) were identified as capable of fitting Wild Type data. To refine this large group, we relied on the principle of parsimony, preferring simple networks (i.e. those with fewer connections). Most of the acceptable networks were nested (i.e. contained simpler acceptable networks plus additional connections). From these, we identified five parsimonious variants containing no simpler acceptable networks. We additionally included two networks with extra connections, chosen arbitrarily, to provide a comparison for trade-offs in more complex, but uninformed, models. (‘Alt6’, ‘Alt7’). We compared these networks against the Core, using all available data to generate a Pareto front for each.

**Figure 5:**
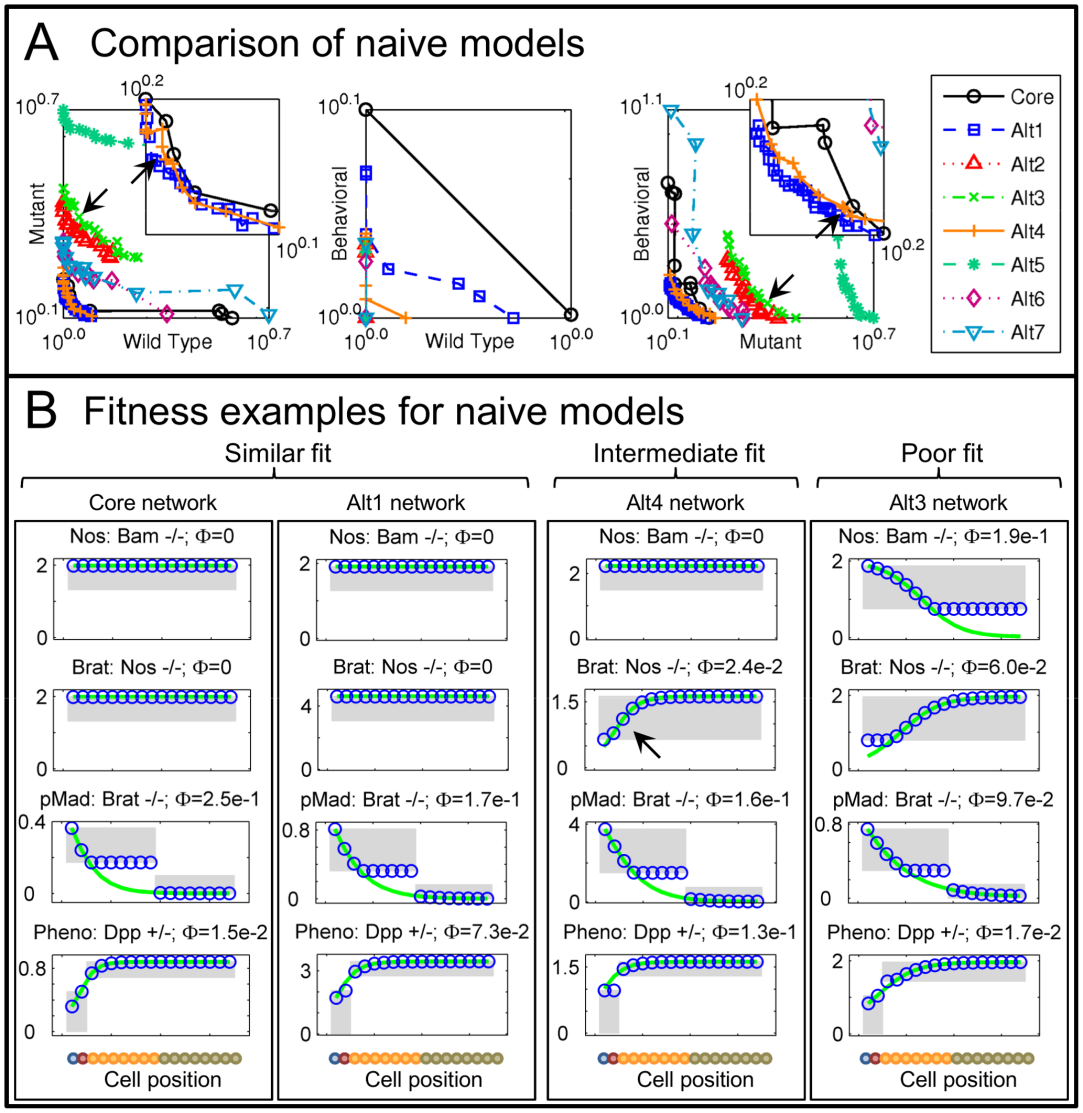
Naive network comparison. A) Solved Pareto fronts for naively generated networks, superimposed for comparison. Insets enlarge view near Utopian point. B) Examples of model fitness, comparing the Core network with the two closest alternatives (based on Pareto front placement) and a poor alternative. Only Alt1 remains similar to the Core on examination. Simulated from Pareto points closest to the Utopian, black arrows in A. Notation as in Figures 3 and 2A.

Pareto fronts determined for each of the alternative networks are shown superimposed in Figure 5A (between 11 and 46 Representatives per network). All networks fail to fully satisfy the data. Examining the Wild Type vs. Mutant projection to compare performance among networks, the Core network dominates most alternatives (Figure 5A, left). However, networks Alt1 and Alt4 perform very similarly to the Core, dominating it at some points. To more closely compare these three models, we examine fitness to individual data (Figure 5B, plots from the Representatives nearest the Utopian, arrows in Figure 5A). For reference, we also present results from Alt3, which performs poorly (e.g. compare top plots, where Nos is observed uniformly high in Bam mutants). In the Nos mutant where Brat data are uniformly high, Alt3 fails while Alt4 performs quantitatively better (Figure 5B 2nd row). However, the qualitative decrease in the anterior region for Alt4 indicates that its structure is less consistent with the mutant phenotype than the Core or Alt1 (compare 2nd plots for each network, decrease in Alt4 indicated by arrow).

The Core and Alt1 networks perform quite similarly, with only minor differences among the unfit data (Figure 5B, compare 3rd and bottom plots). All of the networks compared failed to fit these data. The similar performance of these two networks is explained by similarity in structure. The only difference between the two is that Alt1 lacks feedback of Brat upon Mad (Figure 4A). The basic structure of the Core network is thus well supported, but the data provide poor support for the feedback component.

These comparisons and the relative lack of support for feedback exemplify how sparse and qualitative data can be limiting, even when evaluated quantitatively. Rather than suggesting that the well observed feedback element is not involved, this study indicates that the readily available data from genetic experiments are not sensitive to feedback on Mad. Instead, biochemical evidence indicates the repression of Mad in the presence of Brat (with Pumilio as a cofactor) in a *Drosophila* S2 cell line [12]. While such data can be applied directly to define a model, it is not an explicit observation of the germarium that can be compared to simulations. Furthermore, to better understand the system and build parsimonious models, we encourage considering feasible alternatives to the observed interactions, and asking what is necessary for the system to function, i.e. if elements are indispensable, redundant or unimportant. The example experiment design provided below suggests other genetic experiments in the germarium that may be more sensitive to the feedback on Mad.

### Data do not discriminate more complex hypothesized networks

**Figure 6:**
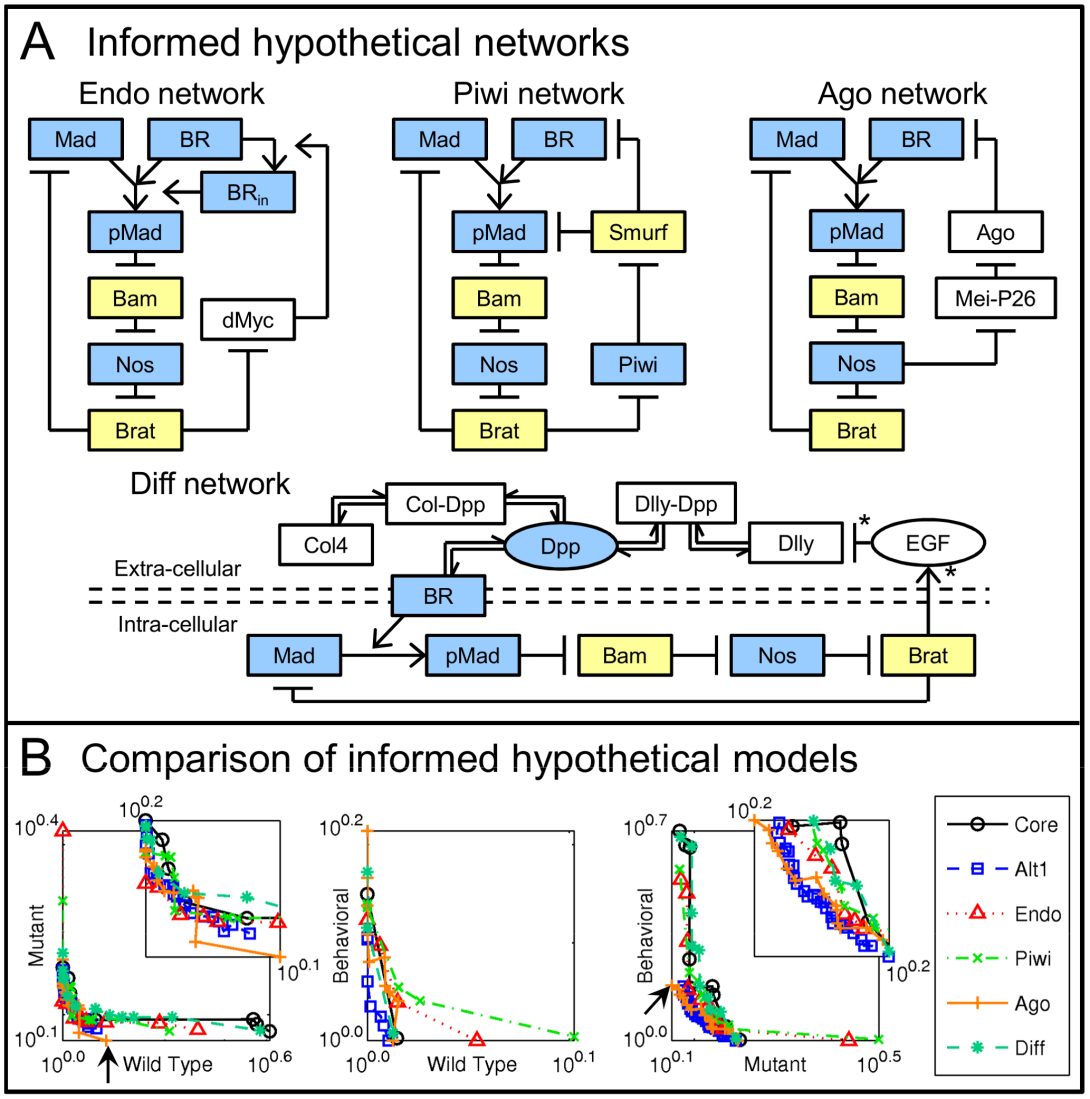
Informed network comparison. A) Informed hypothetical networks, each adding elements to the Core network. * indicates an indirect interaction, potentially involving many intermediaries. Coloring as previously, with white boxes where effects are not conceptually clear. B) Solved Pareto fronts for informed hypothetical networks, including Alt1 which performs similarly to the Core. Arrows indicate the only distinguishable feature among them, where Ago1 improves over other networks, though only at one point.

We constructed four hypothetical networks that include additional regulatory mechanisms, as discussed in recent literature. Each contains the Core network along with additional components and interactions (Figure 6A). For simplicity with the current model structure, we do not consider mechanisms based on cell-cell contact and adhesion.

1. The endocytosis mediated network Endo introduces feedback on the cellular endocytosis rate, via Brat inhibiting dMyc production, as previously observed [12]. dMyc promotes endocytosis [15], [16], which hypothetically causes a stem cell to internalize ligand-receptor complexes more quickly, creating competition for ligand.
2. The Piwi mediated network Piwi includes feedback via Piwi and the associated piRNAs, hypothesized to repress Smurf [26], a ubiquitin protein ligase, which promotes the degradation of both pMad [25], [27], [28] and cell-surface receptors for Dpp [29], [30] (in conjunction with an unmodeled cofactor, Fused). Piwi is repressed in Bam expressing cells [31], though direct interaction with Bam has not been investigated. Because Piwi is in the Argonaute family [32], whose members in several organisms associate with proteins bearing TRIM-NHL motifs (e.g. Brat, Mei-P26 in *Drosophila* and TRIM32 in mice) [14], [33], this model places Piwi as a hypothetical target of Brat.
3. In the Argonaute mediated network Ago, feedback is hypothetically mediated by the observed Mei-P26 repression of miRNA levels (interacting with Argonaute-1) [14], taking effect through miR-184, which represses a Dpp receptor as well as pMad signaling [34]. Not all TRIM-NHL protein interactions involve degradation activity [35], so Argonaute-1 itself may not be directly regulated by Mei-P26. However, for simplicity in this network, Ago represents the overall function of Argonaute-1 and miR-184 and is regulated by Mei-P26.
4. The diffusion mediated network Diff regards extracellular modification of effective Dpp diffusivity through its association with both collagen and the proteoglycan Dally [17]–[19]. Germline cells are hypothesized to regulate the expression of Dally in nearby somatic cells through endothelial growth factor (EGF) ligands [20]; herein, the regulation of EGF is placed downstream of Brat, as the hypothetical regulator of differentiation processes. Dpp association with either collagen or Dally limits its diffusivity, with Dally expression modifying the pool of binding sites to retain Dpp nearby a given germline cell.

We include both the Core and Alt1 networks in the analysis, as they perform nearly indistinguishably. Pareto fronts are presented superimposed in Figure 6B. As indicated by the overlap of all fronts, no clear improvements are made by the hypothetical networks, based on the data at hand. The only indication of improved fitness is a lower error achieved by the Ago model for Wild Type and Behavioral data, while relatively well fit to Mutant data (examine left and right plots, respectively, at the Mutant anchor point indicated by arrows). However, no clear improvements are apparent in individual outputs for the Ago model (data not shown). The lack of clear discrimination among models indicates that the currently available data is inadequate to distinguish the expanded mechanisms tested.

### Analysis of hypothetical networks for future development

Beyond model performance, we use the identified Representatives (Pareto points) to assess the relative influence of each observation and parameter as we consider future model development. We examine the distribution of model error to identify which observations are not yet consistently satisfied, and the distribution of parameter sensitivity to identify influential parameters.

#### Few data remain poorly fit

Data that are consistently well fit across all models need no further attention (unless quantitative measurements become feasible). A useful alternative perspective is that these data are not capable of distinguishing between current and more complex models. Consistently well fit data include indices 5, 7, 8, 13, 14, and 17 from Table 1: the phenotype, Nos or Brat in Bam −/−, pMad in dMyc overexpression, Brat in Nos −/−, and the phenotype for Dpp −/−. The few data consistently unfit, which may prove useful in considering future model additions include 11, 15 and 18 in Table 1: pMad in dMyc −/− with dMyc overexpression, pMad in Brat −/−, and the phenotype in Dpp +/−. The remaining data were involved in the tradeoff between Wild Type and Mutant objectives, and will remain useful when evaluated simultaneously. The error distributions examined are available in Supporting Information (Figures S2–S8 in Text S1).

#### Regulatory parameters exert the greatest effect

We use local sensitivity analysis to measure the impact of individual model parameters, though it is specific to each Representative. As expected, the half-maximal concentrations characterizing regulatory interactions produce the most significant effects and are heavily involved in the trade-off among data objectives. Also indicated as important are phosphorylation kinetics for Mad, and the diffusion, binding and degradation of Dpp. Degradation of regulators and dissociation of Dpp from receptors rarely have a significant effect. Distributions of parameter sensitivity are available in Supporting Information (Figures S9–S14 in Text S1).

### Experiment design

Using the Representatives, we are able to perform a simple model based experiment design, aiming to estimate the most informative experiments from a set of hypothetical perturbations and measurements. Each Representative of each model produces an individual estimate of the system response in a novel experiment. Potential experiments can then be selected to reduce uncertainty in model parameters, in model outputs or to discriminate among competing model structures.

**Figure 7:**
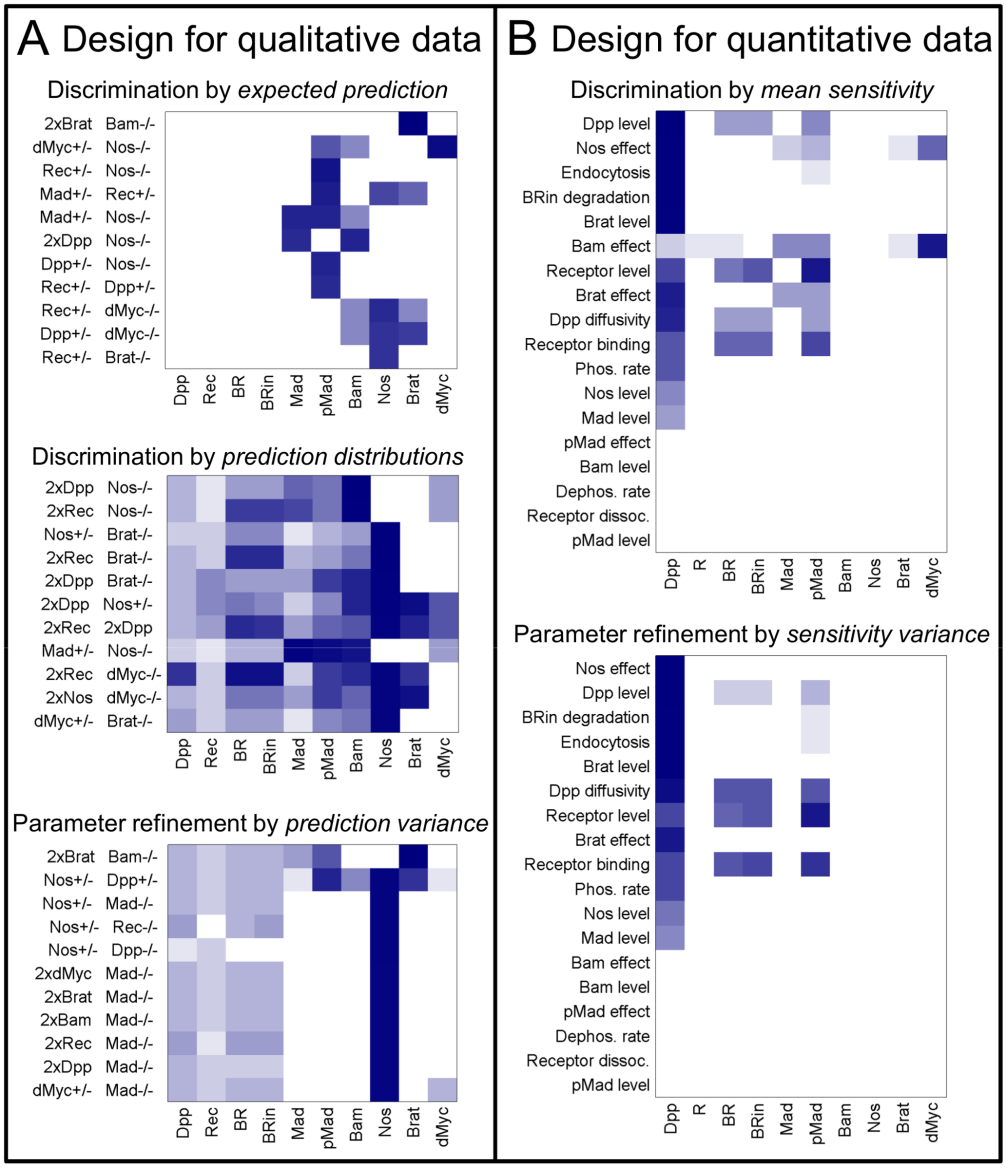
Experiment design. A) Heatmaps showing relative information gain expected from qualitative data, with experiments on the ordinate and species to measure on the abscissa. Darker boxes indicate greater information (i.e. a more preferable experiment), via expected prediction variance among models (upper), dissimilarity of Representative prediction distributions (center), or variance among Representatives (lower). B) Heatmaps showing experiment design for quantitative data. Based on local sensitivity to a parameter affecting the indicated system feature (ordinate), and ranked by variance among models of mean sensitivity (upper) or variance among Representatives (lower).

To consider different expectations from data as well as different modeling goals, we present a small variety of approaches to the experiment design problem. First, we focus on a realistic case, expecting qualitative protein distributions, as with current data. Second, we consider a more ideal scenario expecting quantitative distributions of protein concentration. In each, we rank experiments by their utility in discriminating among models and contrast with a ranking focused on refining parameter estimates. In all cases, we correlate utility with variance of the predicted observations, either among models or Representatives, as greater differences are more likely to be discernible. This is an approach implemented previously [36], [37], also known as a Maximally Informative Next Experiment [38] and satisfying G-optimality [39]. To illustrate the rankings, the top experiments in each design are presented by heatmaps in Figure 7, color intensity indicating the relative information gain expected, based on the objective (e.g. variance with parameters, for reducing uncertainty). Refer to Methods for details on the experiment design procedure and calculation of objectives. For each design, the landscape of objective values over all of the experiments considered exhibits a sharp peak, indicating the importance of carefully selecting the experiment (Supporting Information, Figures S15–S17 in Text S1).

**Figure 8:**
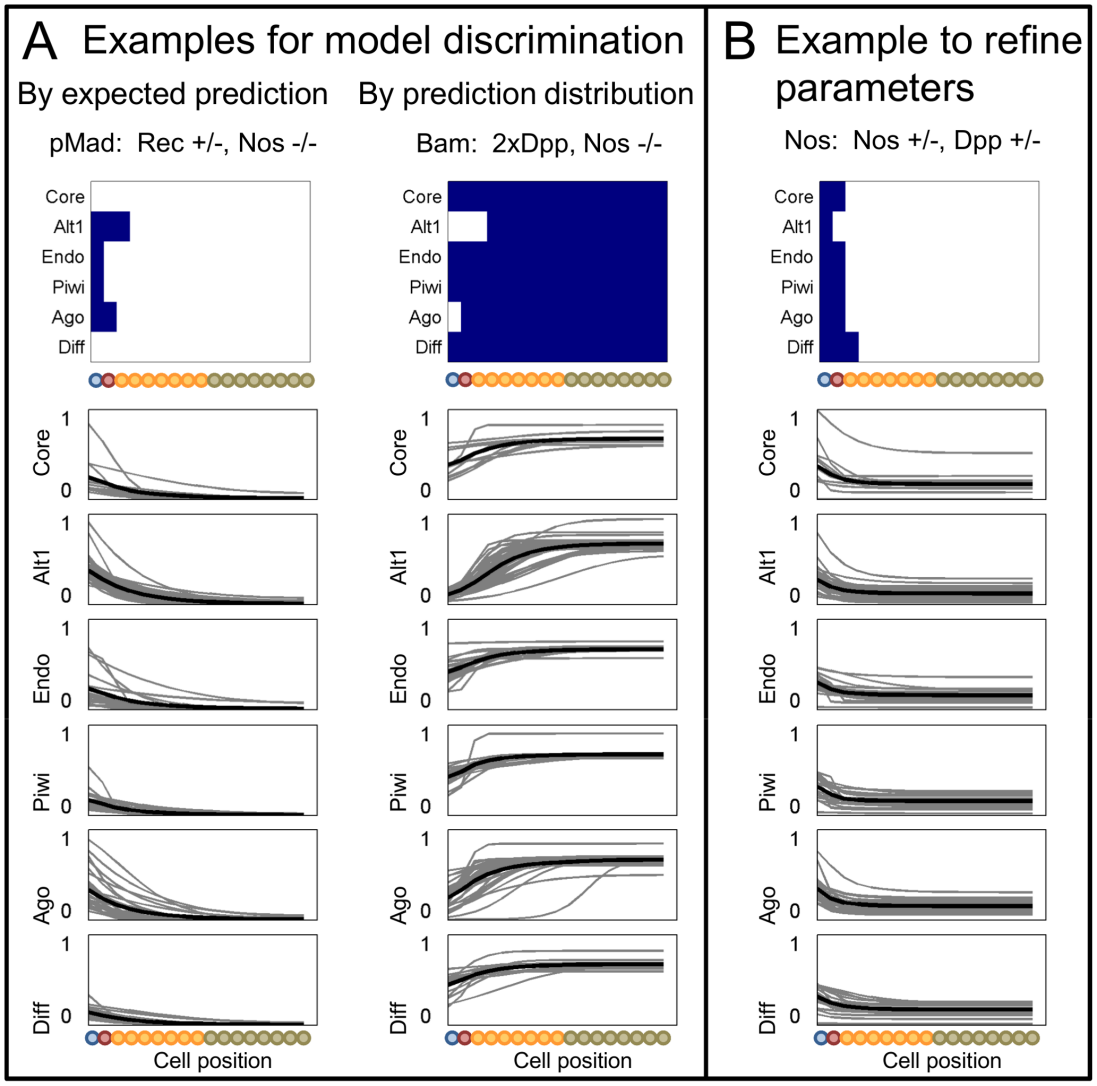
Simulated experiments designed for qualitative data. Example simulated results for recommended experiments, showing for each model: qualitative interpretations (upper) and quantitative model outputs for all Representatives, normalized by the mean value to visualize all curves (lower). A) Simulations for experiments recommended directly for model discrimination. B) Simulations for an experiment recommended to first refine acceptable parameter estimates in each model.

Selected experiments from the designs for qualitative data are shown in Figure 8, where upper panels display expected qualitative predictions and lower plots provide predictions from all Representatives for each model, normalized for visibility. Note that these experiment designs represent a limited range of feasible experiments in this system. More exhaustive model based experiment design carries the promise of more finely resolving system function (e.g. by considering experiments beyond basic genetic perturbations), but is beyond the scope of the current work.

#### Experiment design for qualitative data emphasizes regulators in double mutants

To compare experiments for qualitative data, we simulated each experiment and translated outputs into an expected qualitative observation of high or low concentration. We included single and pairwise combinations of mutation, heterozygous mutation, and doubled genomic content (indicated by 2×) for each of the proteins common to all networks: Dpp, Receptors (Rec), Mad, Bam, Nos, Brat, and dMyc, a total of 210 experiments.

To evaluate model discrimination power, we first ranked experiments based on their *expected* qualitative prediction (Figure 7A, upper heatmap). The *expected prediction* is the most likely observation predicted by a model, with Representatives weighted equally. For these binary predictions, this corresponds to the median value. Figure 8A left shows the predictions for an experiment measuring pMad in Nos −/− Rec +/− (3rd in upper design of Figure 7A). While the expected predictions (upper panel) and the mean predictions (lower plots, black lines) vary somewhat among models, much more variance is evident among the Representatives (gray lines) for each model. We expect the experiment to refine parameter estimates, but not clearly discriminate among models. The two best valued experiments return predictions of uniform distributions of Brat and dMyc in 2×Brat Bam −/− and dMyc +/− Nos −/− respectively, varying only in the predicted level. However, the levels predicted for the Brat experiment also vary more widely within each model than among models, indicating that the experiment is unlikely to discriminate clearly. Conversely, the levels for the dMyc experiment are predicted to be consistently high in all models, except Endo where they are variable and low. Accordingly, this experiment is expected to distinguish the Endo model from the rest, but provide no information on the others. Predictions for these two experiments are available in Supporting Information (Figure S18 in Text S1).

A drawback to considering expected predictions is that they do not consider how individual predictions are *distributed*, or how much they overlap among models. To explicitly consider the overlap of *prediction distributions*, we employ the Jaccard index, which measures the similarity in membership of two sets [1], [40]. It is defined in a pairwise fashion, so we rank experiments based on the sum of all pairwise indices among models. A zero rank would indicate that all models predict the same set of outcomes, while the maximum value would mean that all model prediction sets are mutually exclusive. This design ranks experiments differently than by expected predictions, though some experiments appear in the top set of both (Figure 7A, center). The top experiment, measuring Bam in 2×Dpp Nos −/− (appearing 6th for design by expected prediction), is shown in Figure 8A right. Predictions still vary widely within each model. The expected predictions are less diverse than in the design focused on them (upper heatmaps), and the groups of predictions appear more diverse among models (lower plots, gray lines). Examining the prediction distributions however, we expect little clear discrimination except between Alt1 and the rest (i.e. adding support for the Core feedback element).

As the predicted power to discriminate among models is limited by the uncertainty within each model, better identifying parameters for each model should improve the ability to discriminate. To this end, we design to reduce parameter uncertainty by evaluating the *variance* among *predictions* from the Representatives in each model (Figure 7A, lower). The top experiment corresponds with that from the expected predictions (Brat in 2×Brat Bam −/−). Predictions for the 2nd experiment, Nos in Nos +/− Dpp +/−, show wide variance among Representatives, with little difference among models in either mean values or distributions (Figure 8B).

Based on these predicted simulations, we expect that a combination of experiments designed to reduce parameter uncertainty and to subsequently discriminate will be most effective. While beyond the scope of this work, design for parallel experiments is a promising approach to more reliably estimate the best set of experiments without performing each sequentially [37], [41]. For initial experiments, we recommend working to refine model parameters. However, we caution that predictions for spatially uniform data are subject to uncertainty in the sensitivity of the assay used. Accordingly, we recommend experiments predicted to produce non-uniform results, such as measuring Nos in Nos +/− Dpp +/−. It is also worth considering that the measurement of Bam in 2×Dpp Nos −/− and of dMyc in dMyc +/− Nos −/−, as they are expected to test the Alt1 and Endo models, respectively (Figure 8A right, and S18 in Text S1).

#### Experiment design for quantitative data emphasizes Dpp

To evaluate experiments anticipating quantitative data, we took a classical approach [14] and evaluated the local sensitivity of model outputs to parameters, for each Representative point. However, our approach differs from classical Fisher Information Matrix based optimal designs in that we consider either model discrimination or uncertainty among multiple Representatives, rather than uncertainty around a single parameter set. We define experiments as the choice of an output to measure and a perturbation related to a model parameter. As with the designs for qualitative data, we evaluate experiments both for discrimination and to refine parameters. To assess discrimination, we rank experiments by the variance of *mean sensitivity* among models (Figure 7B, upper). For parameter refinement, we calculated the *variance* of *sensitivity* over Representatives, summing over models (Figure 7B, lower). Recognizing that the model parameters may be affected in multiple ways in the real system, the experiments are listed by the general model feature that is perturbed.

Both designs emphasize measurement of Dpp concentration, which is not expected to be informative in the design for qualitative data. Notably, pMad is the next most useful measurement predicted to refine parameters, and should be considered as well. The two designs differ only slightly in the rank of experiments, indicating little difference between refining parameters and discriminating models.

In this case, because we have employed local sensitivity analyses, we are designing for experiments that perturb the system only slightly. While desirable to limit side effects, this is difficult to implement for internal components in most biological systems. To robustly design for experiments that more significantly perturb conditions, more explicit predictions may be simulated, as with the qualitative design. It is also important to note that we have not considered expected experimental error (i.e. if the predicted results would be distinguishable from the noise). There are several alternative approaches to model based experiment design which may be applicable, depending on the scope and state of the model. For more detail and instruction in experiment design for complex systems, we recommend recent reviews and contributions [41]–[43].

In considering all designs, it is important to also consider the feasibility of experiments, and any alternative means of acquiring similar data. For these example designs, we have included perturbations of all major system components despite the fact that some may be difficult to produce or to evaluate in a real organism. If such experiments are ranked highly, alternative experiments may be necessary to more practically deliver similar information. For example, some genetic mutants may be lethal or may severely disrupt organism development. However, site specific recombination methods or clonal mutation may be able to provide the relevant information without affecting the entire system as drastically. In such cases, it is also important to properly represent the conditions of the experiment, so models may need to be adapted accordingly.

### Conclusions

In this study we have presented a quantitative model analysis based on qualitative data, via multi-objective optimization with Optimal Scaling fitness estimates. Through our analysis of stem cell regulation in the *Drosophila* germarium, we have demonstrated the estimation of a set of representative parameter sets, discrimination among multiple models, and model-based experiment design.

Using the newly developed process to study the germarium, we have shown the extent to which the existing data employed can discriminate among hypothetical regulatory mechanisms. Current qualitative mRNA and protein image data support the serial inhibition of the (previously presented) Core network, but not the feedback element, which is well evidenced in biochemical data. These data do not distinguish among the more complex mechanisms proposed. Toward future modeling, we indicated data that have yet to be satisfied, model parameters that influence fitness, and presented an example experiment design to improve model discrimination. Based on the limited discrimination expected in the experiment designs performed, we recommend first aiming to reduce parameter uncertainty, e.g. by measuring Nos in Nos +/− Dpp +/−. We also recommend pursuing quantitative measurements for Dpp or pMad, as feasible. The designs presented also indicate a variety of other potential experiments. Beyond these initial experiments however, we recommend a more thorough experiment design with careful attention to the feasibility and cost of different experiments.

The framework we have developed offers benefits in a wide range of applications. In principle, it is appropriate for any mathematical modeling problem where some or all data are limited to qualitative observations. Naturally, there is particular potential for gains in biological applications, where highly complex systems are prevalent. With the *Drosophila* germarium as a prime example, developmental biology presents many potential applications as it focuses on pattern formation and spatio-temporal behavior, as in the organization of body axes, limbs, and organ structures [44]. In the broader context of biology and medicine, a variety of fields exhibit similar problems and may also benefit from more widespread use of qualitative data in mathematical modeling studies such as this one. General examples include mechanobiology [45], neurobiology [46], [47], and tissue engineering [48]. We would like to emphasize that the techniques developed in this work accommodate uncertainty in data. If all data can be taken in a rigorously quantitative format, the Optimal Scaling procedure is unnecessary. We anticipate that these techniques will be most valuable when including historical data and when employing new measurements that are not yet refined enough to ensure quantitative reporting.

## Methods

### Data compilation

The aggregate dataset of observations on the anterior germarium was assembled from published literature only. Sources were identified by a primary search of combinations of the terms Drosophila, germarium, GSC, bam, brat, nos, and mad. Searches were performed via the search engine Google Scholar and the databases Medline, PubMed, and Science Citation Index. A secondary search identified additional data sources from references within and articles citing the primary findings. Sources were screened for experiments and relevant data.

Data are recorded under a variety of conditions, including genetic mutation and overexpression. Some data were excluded to limit the computational cost of simulations, especially from overexpression studies (e.g. expression via the yeast Gal4-UAS system [49]) where the increase of expression over wild type is highly uncertain and requires optimization of experimental parameters. Qualitative data were defined by subjective (visual) review of figures and by the interpretations presented by the original authors (e.g. pMad expression is ranked high in a region because its image intensity there appears clearly greater than elsewhere in the same image, with deference to any declared observations made in the published text). Data repeated in multiple works were included one time in the aggregate set, as the observation best representing the consensus from the field. Many data were recorded via fluorescent immunochemistry, which can be ratiometric (i.e. linearly related to the protein concentration) and is often used quantitatively after normalization. However, it is important to consider that the quality of data relies on the entire experiment, not just the final measurement type. The linearity of the data, which is required to reliably normalize, cannot be assured without express guarantees both that the experimental reaction steps were designed to preserve a linear relationship and that the images available accurately present the original intensity values. Many of the experiments aggregated for this study employed enzyme linked visualization assays not originally intended for quantitative comparison or modeling, so controls were not presented to ensure that the reactions remained linear. In addition, the germarium is composed of a soft tissue with a high degree of geometric variability between images, limiting the ability to combine multiple images by geometric registration and evaluate measurement uncertainty. Accordingly, all data were treated as ordinal, which reflects the subjective evaluations presented in the source literature.

To correlate the Phenotype data to Brat expression, we evaluate the mean Brat concentration over the past 6 hours (expecting unmodeled delays, and a cell cycle less than 24 hours [50], [51]). Accordingly, data observed with a fusome are assigned a higher rank than those with a spectrosome.

### Mathematical modeling

Models of the anterior germarium were designed to represent the system as presented in Figure 1A (see Figure S1 in Supporting Information, Text S1). The models consider secretion of Dpp into the extracellular space, diffusion, receptor binding, and protein levels within each cell, according to the internal regulatory network. Alternative models only differ in the intracellular regulatory network, with the exception that the Diff model includes a secreted molecule not modeled otherwise.

#### Assumptions and implementation

To form the simplest models appropriate for the system and available data, we apply a set of general assumptions, including: (1) well-mixed conditions, (2) simple saturating regulation, (3) cofactor sufficiency, and (4) one-dimensional organization. 1) Solutions within and near each cell are assumed to be homogenous, i.e. that local diffusion is sufficiently fast for the apparent reaction concentration to be equivalent to bulk concentration. Long-range diffusion, over multiple cell diameters, is explicitly modeled. 2) All protein production regulatory processes, both transcriptional and translational, are approximated by a Hill equation with a cooperativity coefficient of two. The Hill equation provides saturating effects scalable by the half-maximal concentration of the repressor. 3) All cofactors required for reactions are assumed to be present and non-limiting. 4) Geometric effects in directions other than along the anterior-posterior (long) axis of the germarium are assumed to be negligible, given the quality and resolution of the available data.

Corresponding with the assumptions made, we formulate models with ordinary differential equations (ODE), representing a one dimensional line of cells oriented along the anterior-posterior axis. Each modeled cell comprises an ODE compartment with state variables for the regulatory and signaling molecules. The formulation for intracellular regulators follows the example model equations (1) and (2). Therein, 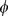 are production rates, 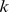 first order degradation, 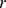 reaction rates, 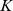 half-maximal concentrations for regulators, and 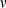 the Hill coefficient (2 throughout all models).
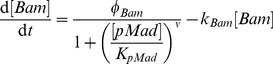
(1)


The model geometry includes the CC at the anterior end to make 18 cells. One cell each is allocated to the wild type GSC and CB positions, the following 7 cells as Cyst, and the last 8 cells as Posterior (as in Figure 1A, bottom). Interconnections among cyst cells are not explicitly modeled. Long range diffusion is approximated by the finite difference method, as in (2).
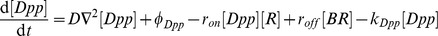


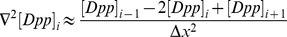
(2)


We apply no-flux boundaries (i.e. 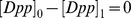 and 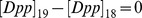), define no Dpp production outside CCs (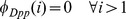), and no Dpp Receptors in the CC (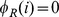). The complete set of equations, as well as model constants and parameter ranges based on literature [12], [16], [22], [52]–[55], are available in Supporting Information (Text S1).

To implement numerically, the ODEs are coded and solved in MATLAB, using the built-in stiff solver ‘ode15s’. For steady state solutions, ODEs are solved from null (zero value) starting conditions over a simulation period of 24 hours. For dynamically constrained solutions, ODEs are first solved for steady state; then a cell cycle and displacement event is approximated and the results used as initial conditions for a 12 hour simulation. Cell cycle and displacement are approximated by setting each zone (GSC, CB, Cyst, Posterior) to the average solved conditions of its anterior neighbor (i.e. shifting values posteriorly by one zone).

#### Alternative network identification

To naively choose networks, we performed two parameter screens for networks that satisfy the available Wild Type data. As all Core regulation is inhibitory, the first screen considered only inhibitory interactions, and was performed by exhaustively sampling combinations of strong and weak regulatory parameters for all possible interactions, excluding self-regulation. Second, to address positive feedback, we performed global optimization over a full range of negative and positive feedback interactions. Both screens were filtered by a fitness threshold and the remaining networks were filtered for parsimony. Further parameter screening details are available in Supporting Information (Text S1).

### Optimal scaling fitness estimation

Optimal Scaling constructs a set of surrogate data, which may take any value within the qualitative constraints observed. The Optimal Scaling problem selects surrogate data that minimize error from the model. Because absolute error values may vary with the scale of the model output, we use squared relative errors 
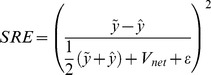
, where 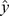 are model outputs, 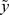 are surrogate data, 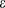 is a small constant to enforce finite values, and 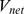 is the net variation across the model geometry, 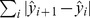 where 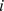 indexes cell position. The inclusion of 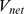 penalizes error in flat model outputs, i.e. common trivial fitness compromises. The final error is the square root of the sum of average error over all cells in each observation domain, 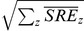, for 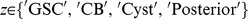.

#### Constraints and implementation

Constraints on surrogate values depend on the data type. For ordinal data and continuous models, the constraints translate to intervals in which each surrogate value must lie (as shown by shaded boxes in Figure 2A), specifically
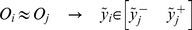


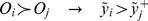
(3)where 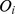 is the 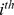 observation, and 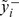 and 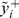 are lower and upper bounds on the interval containing 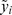. An important implementation note is that surrogate data optimization can be reduced to the selection of interval bounds 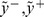. For qualitative data and any given intervals, optimal surrogate values will equal the model output if within the interval, and lie on the nearest boundary if not.

In this application of Optimal Scaling, we estimate constraints on surrogate data interval sizes and spacing based on model values and the resolution of data (i.e. the number of ordinal ranks observed). These constraints reflect that quantitative differences can only be detected over a finite threshold. However, little quantitative information is available on the sensitivity of the experiments considered, so the threshold is unknown for the data at hand. To estimate a generalized constraint, we apply a heuristic based on the scale of model outputs and the number of categories observed in data, 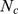. The minimum range of an interval is estimated by 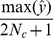; the minimum gap between intervals is limited to 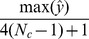. So defined, intervals are prevented from becoming impractically small, while retaining some flexibility by ensuring that a maximum of 75% of the model scale is accounted for by minimum ranges (in the limit as 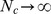, expecting 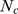 intervals and 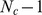 interval gaps).

### Numerical procedures

#### Single objective, global optimization

To screen for the semi-deterministic global optimization, we allocate samples in a deterministic sparse grid (Chebyshev-Gauss-Lobatto node distribution) [56], and pseudo-randomly through a latin hypercube design. The sparse grid provides some sampling uniformity and is also used to define a rough polynomial interpolant, which we use to estimate search start points for multiobjective optimization. Bounded by the computational cost of simulating the model, 500 k samples were evaluated for each screen. Sparse grid density was dependent on the size of the parameter space and was chosen to allocate no more than 75% of the samples deterministically. Gradient searches used the MATLAB built-in constrained optimization routine fmincon, via the interior point method, chosen for strict respect of parameter boundaries. 64 gradient searches were run for each single objective optimization.

#### Multi-objective optimization

We determine the Pareto front using a slightly modified version of normalized normal constraint (NNC) method [24], as depicted in 2C right. Anchor points are determined by the global search, though a single global screen is used for all anchor points. In the normalized space bounded by the Anchor points, valued within 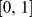 in each objective, the plane including all Anchor points is defined as the Utopian plane. Multiple gradient-based searches are performed, ideally starting from points evenly distributed on the Utopian plane (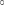 in Figure 2C, right). In order to be more robust to gradient searches settling in local minima, additional search start points on each normal constraint were sought at 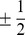 the length of the constraint vector (from zero crossing to the Utopian plane). Initial parameter values corresponding to each desired search start point are estimated by polynomial interpolation on the previously sampled sparse grid [56], rather than by linear interpolation. A gradient search is started from each of these initial parameter estimates (using MATLABs fmincon and the interior point method), requiring that each solution lie on its constraint vector, normal to the Utopian plane. The resulting solutions are filtered for Pareto optimality, returning the final set of Representatives describing the Pareto front.

#### Local sensitivity analysis

We perform local sensitivity analysis by the finite difference method, approximating 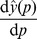 as 
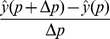
. All sensitivities calculated are relative, i.e. scaled by the nominal value of the output and parameter 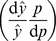.

### Experiment design

Our experiment designs for qualitative data are performed by exhaustively evaluating all of the experiments we consider. To estimate the qualitative observation for each simulation, we apply the surrogate data interval boundary constraints from our Optimal Scaling formulation. These designs then differ only in the objective by which we rank experiments. In all cases, the goal is to maximize the objective value. For each objective, the color intensity plotted in Figure 7 is determined by mapping between RGB colors 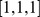 (low) and 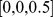 (high), relative to the other values in the same heatmap. Toward discrimination, we rank by the variance over models (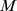) of *expected predictions* (4) or the overlap in *prediction distributions*, using the Jaccard index (5). 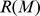 refers to the set of Representatives for model 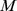. The number of Representatives identified per model varied from 11 to 46. The objective is defined over the 1-D space of the model (17 cells after removing the CC), and we aggregate to a scalar by evaluating the mean of each model region and taking the sum. We define the index 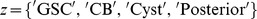 for 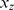, which indicates the cell positions for these regions of the model (as in Figure 1A), as well as 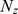 the number of cells in each (i.e. 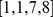).
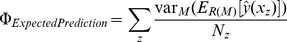
(4)

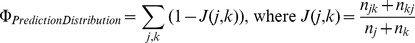
(5)


To calculate the Jaccard index, 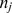 is the number of Representatives in model 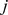, while 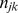 is the number of Representatives of model 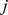 that predict an output also predicted for model 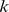, and vice versa for 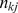. To refine parameters, we rank experiments by the sum over models of the *variance* of *predictions* among Representatives (6).
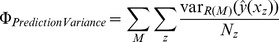
(6)


In the experiment design for quantitative data, we use the local sensitivity results previously discussed, which approximate 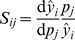. In the objective for discrimination, we aggregate by taking the *mean sensitivity* across Representatives (sensitivities with inconsistent sign will cancel), and rank by variance among models (7). To refine parameters, we evaluate the *sensitivity variance* over Representatives and rank by the sum of this variance over models (8).
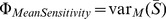
(7)

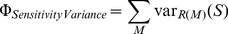
(8)


## Supporting Information

Dataset S1 **Core model representative parameters.** Representative parameter values determined for the Core model, corresponding with Pareto points plotted in Figure 3A, 5A and 6B.
(CSV)

Dataset S2 **Alt1 model representative parameters.** Representative parameter values determined for the Alt1 model, corresponding with Pareto points plotted in Figure 5A and 6B.
(CSV)

Dataset S3 **Endo model representative parameters.** Representative parameter values determined for the Endo model, corresponding with Pareto points plotted in Figure 6B.
(CSV)

Dataset S4 **Piwi model representative parameters.** Representative parameter values determined for the Piwi model, corresponding with Pareto points plotted in Figure 6B.
(CSV)

Dataset S5 **Ago model representative parameters.** Representative parameter values determined for the Ago model, corresponding with Pareto points plotted in Figure 6B.
(CSV)

Dataset S6 **Diff model representative parameters.** Representative parameter values determined for the Diff model, corresponding with Pareto points plotted in Figure 6B.
(CSV)

Text S1 **Supporting Information.** Supporting information regarding model development and analysis.
(PDF)
