# Supplementary material for: Model-Based Analysis for Qualitative Data: An Application in Drosophila Germline Stem Cell Regulation
Source: PLoS Comput Biol. 2014 Mar 13;10(3):e1003498. doi: 10.1371/journal.pcbi.1003498 (PMC3952817; doi:10.1371/journal.pcbi.1003498)
Supplement: Text S1 — Supporting Information. Supporting information regarding model development and analysis. (PDF) [file pcbi.1003498.s007.pdf]

# Model-Based Analysis for Qualitative Data: An Application in *Drosophila* Germline Stem Cell Regulation Supporting Text S1

This supporting text file contains supporting Figures S1-S18, and Tables S1-S3. Datasets S1-S6 are provided as separate files.

## Model development

The models used in this study were based on the 3-D model of the germarium system previously published [12]. To minimize computation time and allow for broad screening and optimization of parameter sets, a 1-D representation of the anterior-posterior axis was discretized, with each node representing a cell. The model was designed as representing two rows of cells to approximate a germarium (i.e. a 2 x 18 array of cells), from anterior to posterior: 2 cap cells, 2 GSCs, 2 CBs, a 2-cell cyst, a 4-cell cyst, a 8-cell cyst, and a 16-cell cyst (Figure S1, color matched to representation in Figure 1 of the main text). Because cells in germaria tend to be more randomly arranged and vary in size and shape, we represent cells as 'squished' in the anterior-posterior (A-P) axis. Germarium length and approximate cell sizes were chosen based on observations [16] and scaled images [12,22,52]. Cell volumes and surface area are calculated assuming an ellipsoid that just fits in each modeled 'box'. Since this representation is symmetric between the two rows, only the 1-D version is necessary. All models are defined by ordinary differential equations (ODE), with a finite difference approximation to calculate diffusive flux among nodes.

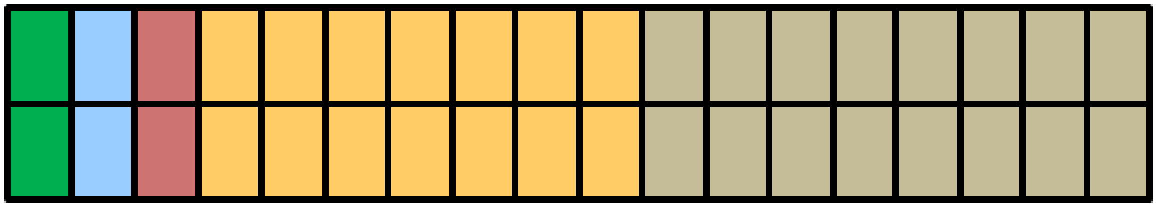

**Figure S1. Basic model geometry.** The 1-D model is equivalent to one row of this 2-D version. Anterior is left.

Model equations are provided here first for the Core model. Additions to and differences from the Core equations are provided for the other model variants. Models of the alternative core networks are not explicitly provided, but all equations match those of the Core model with appropriate changes to regulatory terms. Positive (activating) regulation is in the form, $\phi_Y \frac{\left({[X]}/{K_{X\text{-}Y}}\right)^{\nu}}{1+\left({[X]}/{K_{X\text{-}Y}}\right)^{\nu}}$, for protein $X$ activating protein $Y$. Model variables are defined in Table S1. Constants are defined in Table S2, and parameters are defined in Table S3.

The model equations provided here include the partial differential form of the diffusion terms. Though not explicitly included in the notation, all state variables are function of time, $t$, and position $x$. However, derivatives only depend explicitly on $x$ for diffusing states (Dpp, EGF). As implemented, time is continuous, while position is discretized, representing the cell number

from the anterior, $x \in [1, 2, ..., 18]$. Additional constants were included as needed to reproduce any experimental procedures, such as ectopically or over-expressing a protein.

**Table S1. Model Variables**

| State Variable Name | Symbol | Units |
|---|---|---|
| Core model | | |
| Dpp | $[Dpp]$ | nM |
| Dpp Receptor | $[R]$ | molecules/$\mu$m$^2$ |
| Bound Receptor (Dpp-R complex) | $[BR]$ | molecules/$\mu$m$^2$ |
| Endocytosed Bound Receptor | $[BRin]$ | nM |
| Mad | $[Mad]$ | nM |
| pMad (phosphorylated Mad) | $[pMad]$ | nM |
| Bam | $[Bam]$ | nM |
| Nos | $[Nos]$ | nM |
| Brat | $[Brat]$ | nM |
| dMyc | $[dMyc]$ | nM |
| Piwi model | | |
| Piwi | $[Piwi]$ | nM |
| Smurf | $[Smrf]$ | nM |
| Argonaut model | | |
| Mei-P26 | $[Mei]$ | nM |
| Argonaut-1 | $[Ago]$ | nM |
| Diffusion model | | |
| Epidermal Growth Factor | $[EGF]$ | nM |
| Dally | $[Dlly]$ | nM |
| Dpp-Collagen complex | $[BC]$ | nM |
| Dpp-Dally complex | $[BD]$ | nM |

**Table S2. Model Constants**

| **Constant Name** | **Symbol** | **Value** |
|---|---|---|
| Core model | | |
| Germarium length | - | 50 $\mu$m |
| Cell diameter (normal to A-P axis) | - | 5 $\mu$m |
| Cell volume (ellipsoidal) | - | 36 $\mu m^3$ |
| Cell surface area (ellipsoidal) | - | 56 $\mu m^2$ |
| Surface to (intracellular) Volume conversion | $C_{S2Vi}$ | 2.59 nM/$\frac{\text{molecules}}{\mu m^2}$ |
| Surface to (extracellular) Volume conversion | $C_{S2Ve}$ | 2.85 nM/$\frac{\text{molecules}}{\mu m^2}$ |
| Dpp production rate | $\phi_{Dpp}$ | 5e-4 nM/s |
| Receptor production rate | $\phi_R$ | 4e-4 $\frac{\text{molecules}}{\mu m^2}$/s |
| Dpp expression domain | $\alpha_{Dpp}(x)$ | 1 if $x = 1$<br>0 if$x \neq 1$ |
| Receptor expression domain | $\alpha_R(x)$ | 1 if $x > 1$<br>0 if$x = 1$ |
| Mad production rate | $\phi_{Mad}$ | 5e-4 nM/s |
| Intracellular protein production rate | $\phi_{Bam}, \phi_{Nos}, \phi_{Brat}, \phi_{dMyc}$ | 1e-3 nM/s |
| dMyc degradation rate | $k^{deg}_{dMyc}$ | 1e-3 $s^{-1}$ |
| Cooperativity (Hill) coefficient | $\nu$ | 2 |
| Piwi model | | |
| Intracellular protein production rate | $\phi_{Piwi}, \phi_{Smrf}$ | 1e-3 nM/s |
| Piwi cooperativity coefficient | $\nu_{Piwi}$ | 1 |
| Argonaut model | | |
| Intracellular protein production rate | $\phi_{Mei}, \phi_{Ago}$ | 1e-3 nM/s |
| Argonaut cooperativity coefficient | $\nu_{Ago}$ | 1 |
| Diffusion model | | |
| Collagen total (i.e. binding sites for Dpp) | $Cgn$ | 100 nM |
| Intracellular protein production rate | $\phi_{EGF}, \phi_{Dlly}$ | 1e-3 nM/s |

**Table S3. Model Parameters**

| **Parameter Name** | **Symbol** | **Range allowed** |
|---|---|---|
| Core model | | |
| Dpp degradation rate | $k_{Dpp}^{deg}$ | [2.5e-6, 2.5e-2] $s^{-1}$ |
| Dpp diffusivity | $D_{Dpp}$ | [1e-2, 1] $\mu m^2/s$ |
| Dpp-Receptor association rate | $k_{BR}^{on}$ | [1.1e-4, 4.2e-4] $nM^{-1}s^{-1}$ |
| Dpp-Receptor dissociation rate | $k_{BR}^{off}$ | [2e-4, 8e-4] $s^{-1}$ |
| Receptor degradation rate | $k_{R}^{deg}$ | [4e-8, 4e-4] $s^{-1}$ |
| Endocytosed BR degradation rate | $k_{BRin}^{deg}$ | [2e-6, 2e-2] $s^{-1}$ |
| Intracellular protein degradation rate | $k_{Mad}^{deg}, k_{pMad}^{deg}, k_{Bam}^{deg}, k_{Nos}^{deg}, k_{Brat}^{deg}$ | [1e-7, 1e-3] $s^{-1}$ |
| Mad phosphorylation rate | $k_{Mad}^{phos}$ | [4.8e-6, 4.8e-2] $\frac{\text{molecules}}{\mu m^2}/s$ |
| Mad dephosphorylation rate | $k_{Mad}^{dephos}$ | [1.1e-5, 1.1e-1] $s^{-1}$ |
| Regulatory half-maximal fractions | $K_{pMad}^{frac}, K_{Bam}^{frac}, K_{Nos}^{frac}, K_{Brat\text{-}Mad}^{frac}$ | [0, 1] |
| Endocytosis rate | $k^{e}$ | [4e-6, 4e-2] $s^{-1}$ |
| Endocytosis model | | |
| dMyc degradation rate | $k_{dMyc}^{deg}$ | [5e-4 2e-3] $s^{-1}$ |
| Regulatory half-maximal fraction | $K_{Brat\text{-}dMyc}^{frac}$ | [0, 1] |
| Endocytosis dependence on dMyc | $K_{dMyc}^{e}$ | [0, 1] |
| Piwi model | | |
| Intracellular protein degradation rate | $k_{Piwi}^{deg}, k_{Smrf}^{deg}$ | [1e-7, 1e-3] $s^{-1}$ |
| Regulatory half-maximal fraction | $K_{Brat\text{-}Piwi}^{frac}, K_{Piwi}^{frac}$ | [0, 1] |
| Smurf-mediated pMad degradation rate | $k_{pMad}^{ubq}$ | [1e-9, 1e-5] |
| Smurf-mediated Receptor degradation rate | $k_{R}^{ubq}$ | [4e-10, 4e-6] |
| Argonaut model | | |
| Intracellular protein degradation rate | $k_{Mei}^{deg}, k_{Ago}^{deg}$ | [1e-7, 1e-3] $s^{-1}$ |
| Regulatory half-maximal fraction | $K_{Nos\text{-}Mei}^{frac}, K_{Mei}^{frac}, K_{Ago}^{frac}$ | [0, 1] |

**Table S3. Model Parameters**

| Parameter Name | Symbol | Range allowed |
|---|---|---|
| Diffusion model | | |
| Intracellular protein degradation rate | $k^{deg}_{EGF}, k^{deg}_{Dlly}$ | [1e-7, 1e-3] $s^{-1}$ |
| EGF diffusivity | $D_{EGF}$ | [5e-2, 5] $\mu m^2/s$ |
| Regulatory half-maximal fraction | $K^{frac}_{Brat\text{-}EGF}, K^{frac}_{EGF}$ | [0, 1] |
| Dpp-Collagen association rate | $k^{on}_{BC}$ | [2e-6, 7.8e-6] $nM^{-1}s^{-1}$ |
| Dpp-Collagen dissociation rate | $k^{off}_{BC}$ | [1.5e-3, 5.8e-3] $s^{-1}$ |
| Dpp-Dally association rate | $k^{on}_{BD}$ | [7e-6, 2.8e-5] $nM^{-1}s^{-1}$ |
| Dpp-Dally dissociation rate | $k^{off}_{BD}$ | [8e-3, 3.2e-2] $s^{-1}$ |

Regulatory fractions, $K^{frac}_X$, where $X$ is any of the regulators, are converted into the regulatory half-maximal concentration by scaling with the steady state concentration of the regulator, as in (1). Protein production rates and ranges for degradation rates are generalized to allow wide variation. In the Diffusion model, collagen total concentration is generalized to represent a high availability of binding sites. Dpp production and degradation rates are generalized, but based on observations of Dpp expression in the *Drosophila* wing imaginal disc [53]. Dpp association and dissociation rate ranges are based on observations of the homologue BMP-2 interacting with immobilized BMP-R1A [54]. Mad production, phosphorylation and dephosphorylation rates are based on observations of Smad nucleocytoplasmic shuttling in HaCat cells [55]. The production rate and degradation ranges for dMyc are designed to provide a normalized response in [0, 1] at the mean value, simplifying the implementation of dMyc-mediated endocytosis (i.e. no scaling required for a 0 to 1 relative effect via $K^{e}_{dMyc}$, with some extra flexibility to the maximum endocytosis rate via the dMyc degradation parameter). Ranges for the Smurf-mediated degradation (a ubiquitination process) were selected for a wide range of effects, centered about the value that doubles the degradation rate, for a maximum Smurf concentration, i.e. $k^{ubq}_X = k^{deg}_X \frac{k^{deg}_{Smrf}}{\phi_{Smrf}}, \quad \forall X \in \{R, pMad\}$.

$$K_X = K^{frac}_X \frac{\phi_X}{k^{deg}_X}, \quad \forall X \in \left\{ \begin{array}{l} pMad, Bam, Nos, Brat\text{-}Mad, Brat\text{-}dMyc, Brat\text{-}Piwi, \\ Piwi, Nos\text{-}Mei, Mei, Ago, Brat\text{-}EGF, EGF \end{array} \right\} \quad (1)$$

## Core model

$$\frac{\partial[Dpp]}{\partial t} = D_{Dpp}\frac{\partial^2[Dpp]}{\partial x^2} + \phi_{Dpp}\alpha_{Dpp}(x) - k_{BR}^{on}C_{S2Ve}[Dpp][R] + k_{BR}^{off}C_{S2Ve}[BR] - k_{Dpp}^{deg}[Dpp] \quad (2)$$

$$\frac{\mathrm{d}[R]}{\mathrm{d}t} = \phi_R\alpha_R(x) - k_{BR}^{on}[Dpp][R] + k_{BR}^{off}[BR] - k_R^{deg}[R] \quad (3)$$

$$\frac{\mathrm{d}[BR]}{\mathrm{d}t} = k_{BR}^{on}[Dpp][R] - k_{BR}^{off}[BR] - k^e\Big(1 + K_{dMyc}^e\big([dMyc]-1\big)\Big)[BR] \quad (4)$$

$$\frac{\mathrm{d}[BRin]}{\mathrm{d}t} = k^e\Big(1 + K_{dMyc}^e\big([dMyc]-1\big)\Big)C_{S2Vi}[BR] - k_{BRin}^{deg}[BRin] \quad (5)$$

$$\frac{\mathrm{d}[Mad]}{\mathrm{d}t} = \frac{\phi_{Mad}}{1+\left(\frac{[Brat]}{K_{Brat\text{-}Mad}}\right)^{\nu}} - k_{Mad}^{phos}[Mad]\big([BR]C_{S2Vi} + [BRin]\big) + k_{Mad}^{dephos}[pMad] - k_{Mad}^{deg}[Mad] \quad (6)$$

$$\frac{\mathrm{d}[pMad]}{\mathrm{d}t} = k_{Mad}^{phos}[Mad]\big([BR]C_{S2Vi} + [BRin]\big) - k_{Mad}^{dephos}[pMad] - k_{pMad}^{deg}[pMad] \quad (7)$$

$$\frac{\mathrm{d}[Bam]}{\mathrm{d}t} = \frac{\phi_{Bam}}{1+\left(\frac{[pMad]}{K_{pMad}}\right)^{\nu}} - k_{Bam}^{deg}[Bam] \quad (8)$$

$$\frac{\mathrm{d}[Nos]}{\mathrm{d}t} = \frac{\phi_{Nos}}{1+\left(\frac{[Bam]}{K_{Bam}}\right)^{\nu}} - k_{Nos}^{deg}[Nos] \quad (9)$$

$$\frac{\mathrm{d}[Brat]}{\mathrm{d}t} = \frac{\phi_{Brat}}{1+\left(\frac{[Nos]}{K_{Nos}}\right)^{\nu}} - k_{Brat}^{deg}[Brat] \quad (10)$$

$$\frac{\mathrm{d}[dMyc]}{\mathrm{d}t} = \phi_{dMyc} - k_{dMyc}^{deg}[dMyc] \quad (11)$$

## Model variants

For brevity in presenting the model variants, only the equations that differ from or do not appear in the Core model are provided.

### Endocytosis model

$$\frac{\mathrm{d}[dMyc]}{\mathrm{d}t} = \frac{\phi_{dMyc}}{1+\left(\frac{[Brat]}{K_{Brat\text{-}dMyc}}\right)^{\nu}} - k_{dMyc}^{deg}[dMyc] \quad (12)$$

**Piwi model**

$$\frac{\mathrm{d}[Piwi]}{\mathrm{d}t} = \frac{\phi_{Piwi}}{1+\left(\frac{[Brat]}{K_{Brat\text{-}Piwi}}\right)^{\nu}} - k^{deg}_{Piwi}[Piwi] \tag{13}$$

$$\frac{\mathrm{d}[Smrf]}{\mathrm{d}t} = \frac{\phi_{Smrf}}{1+\left(\frac{[Piwi]}{K_{Piwi}}\right)^{\nu_{Piwi}}} - k^{deg}_{Smrf}[Smrf] \tag{14}$$

$$\frac{\mathrm{d}[pMad]}{\mathrm{d}t} = k^{phos}_{Mad}[Mad]\big([BR]C_{S2Vi} + [BRin]\big) - k^{dephos}_{Mad}[pMad] - k^{deg}_{pMad}[pMad] - k^{ubq}_{pMad}[Smrf][pMad] \tag{15}$$

$$\frac{\mathrm{d}[R]}{\mathrm{d}t} = \phi_R\alpha_R(x) - k^{on}_{BR}[Dpp][R] + k^{off}_{BR}[BR] - k^{deg}_{R}[R] - k^{ubq}_{R}[Smrf][R] \tag{16}$$

$$\frac{\mathrm{d}[BR]}{\mathrm{d}t} = k^{on}_{BR}[Dpp][R] - k^{off}_{BR}[BR] - k^{e}\Big(1 + K^{e}_{dMyc}\big([dMyc]-1\big)\Big)[BR] - k^{ubq}_{R}[Smrf][BR] \tag{17}$$

$$\frac{\mathrm{d}[BRin]}{\mathrm{d}t} = k^{e}\Big(1 + K^{e}_{dMyc}\big([dMyc]-1\big)\Big)C_{S2Vi}[BR] - k^{deg}_{BRin}[BRin] - k^{ubq}_{R}[Smrf][BRin] \tag{18}$$

**Argonaut model**

$$\frac{\mathrm{d}[Mei]}{\mathrm{d}t} = \frac{\phi_{Mei}}{1+\left(\frac{[Nos]}{K_{Nos\text{-}Mei}}\right)^{\nu}} - k^{deg}_{Mei}[Mei] \tag{19}$$

$$\frac{\mathrm{d}[Ago]}{\mathrm{d}t} = \frac{\phi_{Ago}}{1+\left(\frac{[Mei]}{K_{Mei}}\right)^{\nu}} - k^{deg}_{Ago}[Ago] \tag{20}$$

$$\frac{\mathrm{d}[R]}{\mathrm{d}t} = \frac{\phi_R\alpha_R(x)}{1+\left(\frac{[Ago]}{K_{Ago}}\right)^{\nu_{Ago}}} - k^{on}_{BR}[Dpp][R] + k^{off}_{BR}[BR] - k^{deg}_{R}[R] \tag{21}$$

**Diffusion model**

$$\frac{\partial[EGF]}{\partial t} = D_{EGF}\frac{\partial^2[EGF]}{\partial x^2} + \phi_{EGF}\frac{\left(\frac{[Brat]}{K_{Brat\text{-}EGF}}\right)^{\nu}}{1+\left(\frac{[Brat]}{K_{Brat\text{-}EGF}}\right)^{\nu}} - k^{deg}_{EGF}[EGF] \tag{22}$$

$$\frac{\mathrm{d}[Dlly]}{\mathrm{d}t} = \frac{\phi_{Dlly}}{1+\left(\frac{[EGF]}{K_{EGF}}\right)^{\nu}} - k^{on}_{BD}[Dpp][Dlly] + k^{off}_{BD}[BD] - k^{deg}_{Dlly}[Dlly] \tag{23}$$

$$\frac{\mathrm{d}[BD]}{\mathrm{d}t} = k^{on}_{BD}[Dpp][Dlly] - k^{off}_{BD}[BD] \tag{24}$$

$$\frac{\mathrm{d}[BC]}{\mathrm{d}t} = k^{on}_{BC}[Dpp]\big(Cgn - [BC]\big) - k^{off}_{BC}[BC] \tag{25}$$

## Fully connected models

Two forms of a fully connected intracellular model were employed when screening for alternative networks to the Core. One uses binary regulatory direction terms, and one uses a fully continuous regulatory parameter space. Both models consider an expanded set of regulatory interactions, wherein parameters are named as $K_{X\text{-}Y}$, for protein $X$ regulating the production of protein $Y$.

### Binary regulatory direction

To best replicate the regulatory terms used in the Core model, a version was formulated with binary regulatory direction (i.e. additional binary parameters indicate if a regulation is activating or inhibiting). These parameters are denoted $\delta_{X\text{-}Y}$, for the protein $X$ regulating protein $Y$. This model was used to screen the expected case of all interactions being inhibitory. It was also used in additional screening, where the binary direction parameters were varied to simulate positive regulation.

$$\begin{aligned}\frac{\mathrm{d}[Mad]}{\mathrm{d}t} &= \\ &\phi_{Mad}\frac{1+\delta_{pMad\text{-}Mad}\frac{[pMad]}{K_{pMad\text{-}Mad}}+\delta_{Bam\text{-}Mad}\frac{[Bam]}{K_{Bam\text{-}Mad}}+\delta_{Nos\text{-}Mad}\frac{[Nos]}{K_{Nos\text{-}Mad}}+\delta_{Brat\text{-}Mad}\frac{[Brat]}{K_{Brat\text{-}Mad}}}{1+\frac{[pMad]}{K_{pMad\text{-}Mad}}+\frac{[Bam]}{K_{Bam\text{-}Mad}}+\frac{[Nos]}{K_{Nos\text{-}Mad}}+\frac{[Brat]}{K_{Brat\text{-}Mad}}} \\ &- k_{Mad}^{phos}[Mad]\big([BR]C_{S2Vi}+[BRin]\big)+k_{Mad}^{dephos}[pMad]-k_{Mad}^{deg}[Mad] \qquad (26)\end{aligned}$$

$$\begin{aligned}\frac{\mathrm{d}[Bam]}{\mathrm{d}t} &= \\ &\phi_{Bam}\frac{1+\delta_{pMad\text{-}Bam}\frac{[pMad]}{K_{pMad\text{-}Bam}}+\delta_{Bam\text{-}Bam}\frac{[Bam]}{K_{Bam\text{-}Bam}}+\delta_{Nos\text{-}Bam}\frac{[Nos]}{K_{Nos\text{-}Bam}}+\delta_{Brat\text{-}Bam}\frac{[Brat]}{K_{Brat\text{-}Bam}}}{1+\frac{[pMad]}{K_{pMad\text{-}Bam}}+\frac{[Bam]}{K_{Bam\text{-}Bam}}+\frac{[Nos]}{K_{Nos\text{-}Bam}}+\frac{[Brat]}{K_{Brat\text{-}Bam}}} \\ &- k_{Bam}^{deg}[Bam] \qquad (27)\end{aligned}$$

$$\begin{aligned}\frac{\mathrm{d}[Nos]}{\mathrm{d}t} &= \\ &\phi_{Nos}\frac{1+\delta_{pMad\text{-}Nos}\frac{[pMad]}{K_{pMad\text{-}Nos}}+\delta_{Bam\text{-}Nos}\frac{[Bam]}{K_{Bam\text{-}Nos}}+\delta_{Nos\text{-}Nos}\frac{[Nos]}{K_{Nos\text{-}Nos}}+\delta_{Brat\text{-}Nos}\frac{[Brat]}{K_{Brat\text{-}Nos}}}{1+\frac{[pMad]}{K_{pMad\text{-}Nos}}+\frac{[Bam]}{K_{Bam\text{-}Nos}}+\frac{[Nos]}{K_{Nos\text{-}Nos}}+\frac{[Brat]}{K_{Brat\text{-}Nos}}} \\ &- k_{Nos}^{deg}[Nos] \qquad (28)\end{aligned}$$

$$\frac{\mathrm{d}[Brat]}{\mathrm{d}t} =$$
$$\phi_{Brat}\frac{1+\delta_{pMad\text{-}Brat}\frac{[pMad]}{K_{pMad\text{-}Brat}}+\delta_{Bam\text{-}Brat}\frac{[Bam]}{K_{Bam\text{-}Brat}}+\delta_{Nos\text{-}Brat}\frac{[Nos]}{K_{Nos\text{-}Brat}}+\delta_{Brat\text{-}Brat}\frac{[Brat]}{K_{Brat\text{-}Brat}}}{1+\frac{[pMad]}{K_{pMad\text{-}Brat}}+\frac{[Bam]}{K_{Bam\text{-}Brat}}+\frac{[Nos]}{K_{Nos\text{-}Brat}}+\frac{[Brat]}{K_{Brat\text{-}Brat}}}$$
$$- k^{deg}_{Brat}[Brat] \tag{29}$$

### Continuous regulatory parameter space

Because in the binary regulatory direction model, it is difficult to perform gradient-based searches that change regulatory direction, a model was formulated with a continuous regulatory parameter space. In this format, negative parameters represent repression and positive parameters activation, while a zero value provides no regulatory input. To this end, the parameters used in this model are the reciprocal of the previously defined regulatory parameters, $K_{iX\text{-}Y} = K^{-1}_{X\text{-}Y}$ (for $X$ regulating $Y$). To reduce the dimension of the search space, self-regulation was not included.

In the following equations, the $\gamma_X$ terms represent a summation of the possible regulatory inputs with null assumption of some expression (the leading value of 0.5). Cooperativity (the Hill function formulation with $\nu = 2$) is included for each regulator. In the first term of each ODE, the resulting regulatory weight $\gamma_X$ is then mapped to a [0, 1] domain, with the constant $\beta = 0.015$ chosen to scale the points of saturation to be within a reasonable range. Note that $\frac{\gamma_X|\gamma_X|}{\beta+\gamma_X^2}$ maps to [-1, 1], so a constant of 1 is added and the result divided by 2.

$$\frac{\mathrm{d}[Mad]}{\mathrm{d}t} = \frac{\phi_{Mad}}{2}\left(1+\frac{\gamma_{Mad}|\gamma_{Mad}|}{\beta+\gamma^2_{Mad}}\right) - k^{phos}_{Mad}[Mad]\big([BR]C_{S2Vi}+[BRin]\big)$$
$$+ k^{dephos}_{Mad}[pMad] - k^{deg}_{Mad}[Mad] \tag{30}$$
$$\gamma_{Mad} = 0.5 + \frac{sgn(K_{iBam\text{-}Mad})}{\big([Bam]K_{iBam\text{-}Mad}\big)^{-\nu}} + \frac{sgn(K_{iNos\text{-}Mad})}{\big([Nos]K_{iNos\text{-}Mad}\big)^{-\nu}} + \frac{sgn(K_{iBrat\text{-}Mad})}{\big([Brat]K_{iBrat\text{-}Mad}\big)^{-\nu}}$$

$$\frac{\mathrm{d}[Bam]}{\mathrm{d}t} = \frac{\phi_{Bam}}{2}\left(1+\frac{\gamma_{Bam}|\gamma_{Bam}|}{\beta+\gamma^2_{Bam}}\right) - k^{deg}_{Bam}[Bam] \tag{31}$$
$$\gamma_{Bam} = 0.5 + \frac{sgn(K_{iNos\text{-}Bam})}{\big([Nos]K_{iNos\text{-}Bam}\big)^{-\nu}} + \frac{sgn(K_{iBrat\text{-}Bam})}{\big([Brat]K_{iBrat\text{-}Bam}\big)^{-\nu}} + \frac{sgn(K_{ipMad\text{-}Bam})}{\big([pMad]K_{ipMad\text{-}Bam}\big)^{-\nu}}$$

$$\frac{\mathrm{d}[Nos]}{\mathrm{d}t} = \frac{\phi_{Nos}}{2}\left(1+\frac{\gamma_{Nos}|\gamma_{Nos}|}{\beta+\gamma^2_{Nos}}\right) - k^{deg}_{Nos}[Nos] \tag{32}$$
$$\gamma_{Nos} = 0.5 + \frac{sgn(K_{iBrat\text{-}Nos})}{\big([Brat]K_{iBrat\text{-}Nos}\big)^{-\nu}} + \frac{sgn(K_{ipMad\text{-}Nos})}{\big([pMad]K_{ipMad\text{-}Nos}\big)^{-\nu}} + \frac{sgn(K_{iBam\text{-}Nos})}{\big([Bam]K_{iBam\text{-}Nos}\big)^{-\nu}}$$

$$\frac{\mathrm{d}[Brat]}{\mathrm{d}t} = \frac{\phi_{Brat}}{2}\left(1 + \frac{\gamma_{Brat}|\gamma_{Brat}|}{\beta + \gamma_{Brat}^2}\right) - k_{Brat}^{deg}[Brat] \tag{33}$$

$$\gamma_{Brat} = 0.5 + \frac{sgn(K_{ipMad\text{-}Brat})}{\left([pMad]K_{ipMad\text{-}Brat}\right)^{-\nu}} + \frac{sgn(K_{iBam\text{-}Brat})}{\left([Bam]K_{iBam\text{-}Brat}\right)^{-\nu}} + \frac{sgn(K_{iNos\text{-}Brat})}{\left([Nos]K_{iNos\text{-}Brat}\right)^{-\nu}}$$

## Screening procedure

Parameter screening for alternative networks was carried out in two phases. First the binary regulatory direction model was used to simulate networks with only inhibitory interactions. For these simulations, only regulatory parameters were allowed to vary. A complete screen was performed with the ~65k combinations of regulatory parameters, $K_{X\text{-}Y}$, at two values: 0.1 (high effect) and 10 (negligible effect). To consider other combinations of parameter values for the only-inhibitory case, a global search was performed as described in the main text. 250k initial samples were simulated, and 512 gradient searches performed. The resulting local optima were included with the combinatorial screen results and filtered to identify satisfactory networks (for wild type data).

To screen for a broader range of interactions, a limited (randomly chosen) set of combinations for binary regulatory direction parameters were sampled. However, to more reliably search over the possible combinations of inhibitory and activating regulation, the continuous parameter space model was employed. Parameter estimation in this model included all parameters varied in the Core model (as well as the additional interactions). Regulatory terms were allowed to vary between -5 and 5. The model was screened using the global search method as described in the main text, with 500k initial samples and 512 gradient searches. All results were filtered for satisfaction, as with the inhibitory screen.

# Model analysis

## Parameterization

Model parameterization (estimation by global and multi-objective optimization) was performed as described in the main text. For reference, we provide the parameter values for all Representatives reported for the hypothetical model comparisons. These parameter sets are provided as comma separated values (CSV) in Supporting Datasets S1-S6 (for the Core, Alt1, Endo, Piwi, Ago, and Diff models, respectively). In each CSV, the first line consists of parameter names, and all remaining lines contain parameter values in exponential format (e.g. 4.663127e-003).

## Error distribution

The distributions of model error over all Representatives in each model are provided here for reference. These distributions were examined subjectively to infer which observations were consistently satisfied and which were likely involved in trade-offs across the Pareto front. Observations are ordered by the median error. A dashed line at the top of the plot indicates truncation for visibility; any point lying on this line is valued greater than or equal to the value shown.

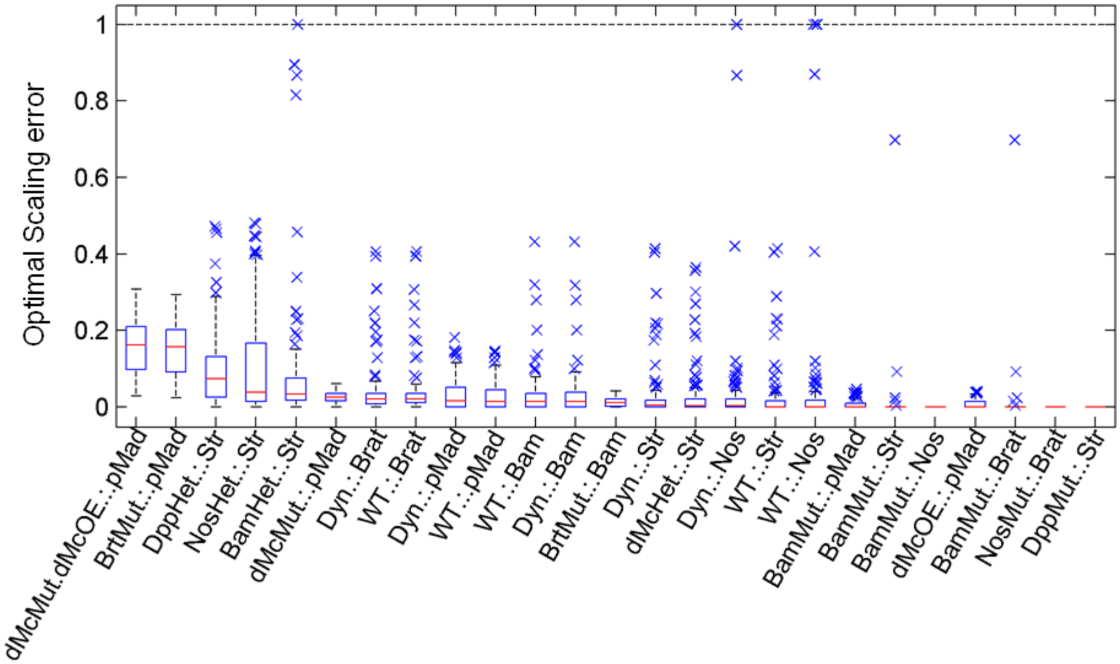


**Figure S2. Distribution of model error over all models.**

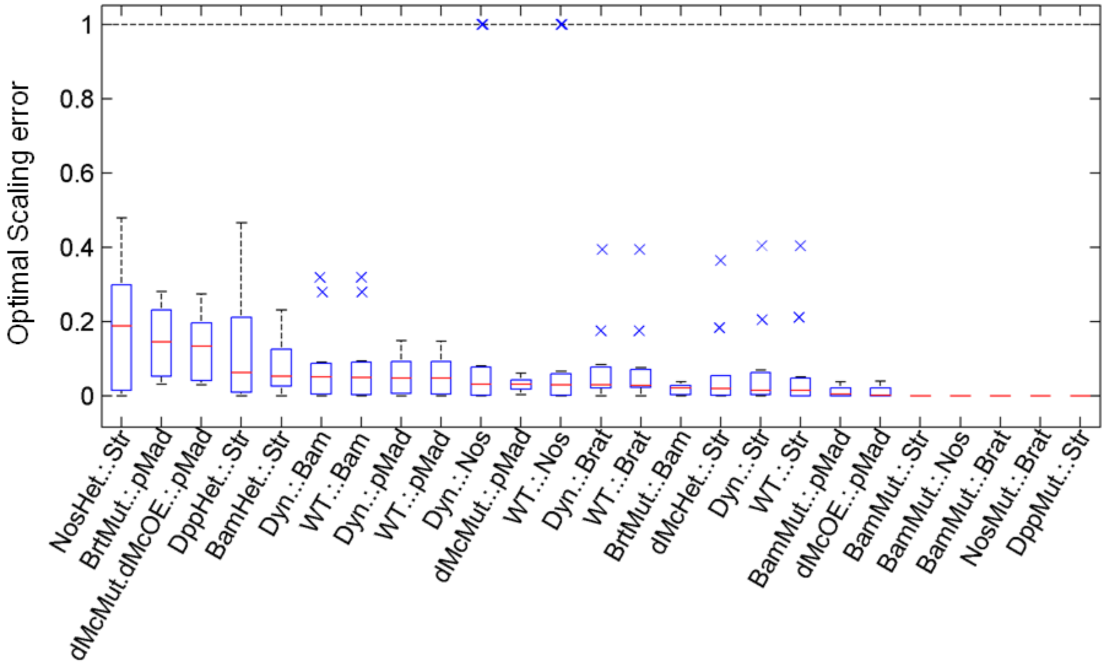


**Figure S3. Distribution of model error over the Core model.**

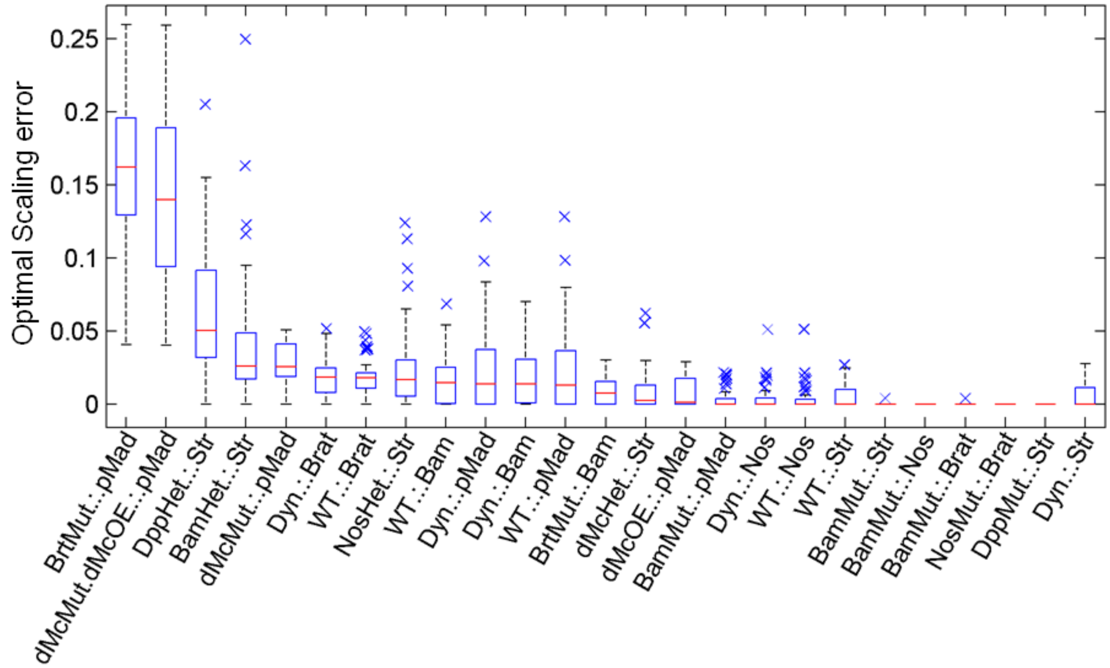


**Figure S4. Distribution of model error over the Alt1 model.**

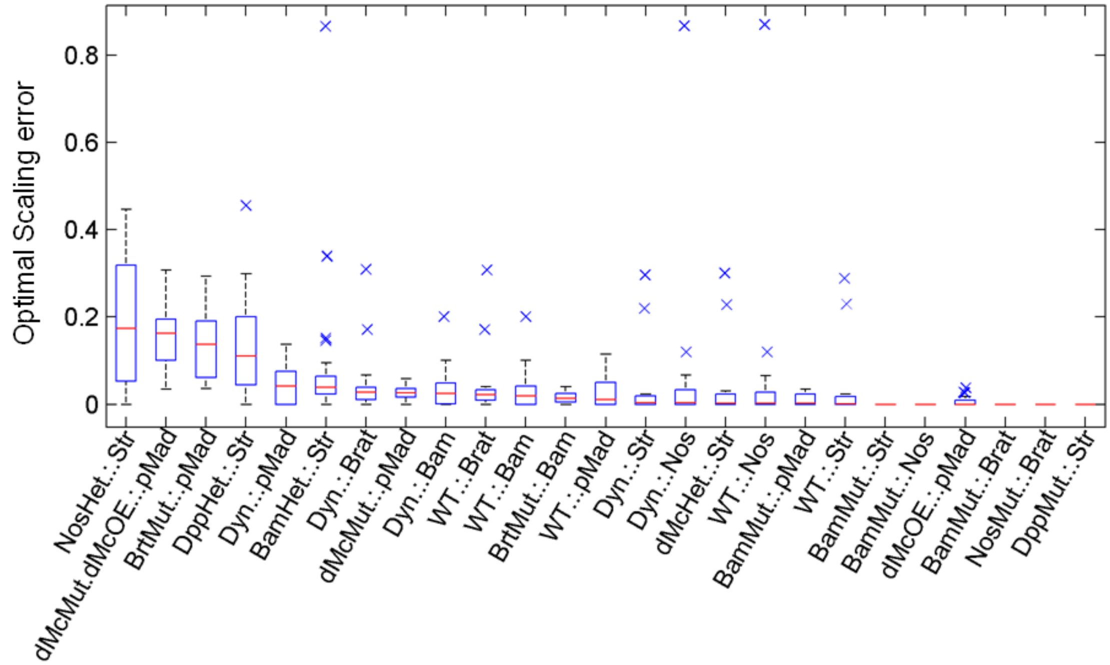


**Figure S5. Distribution of model error over the Endocytosis model.**

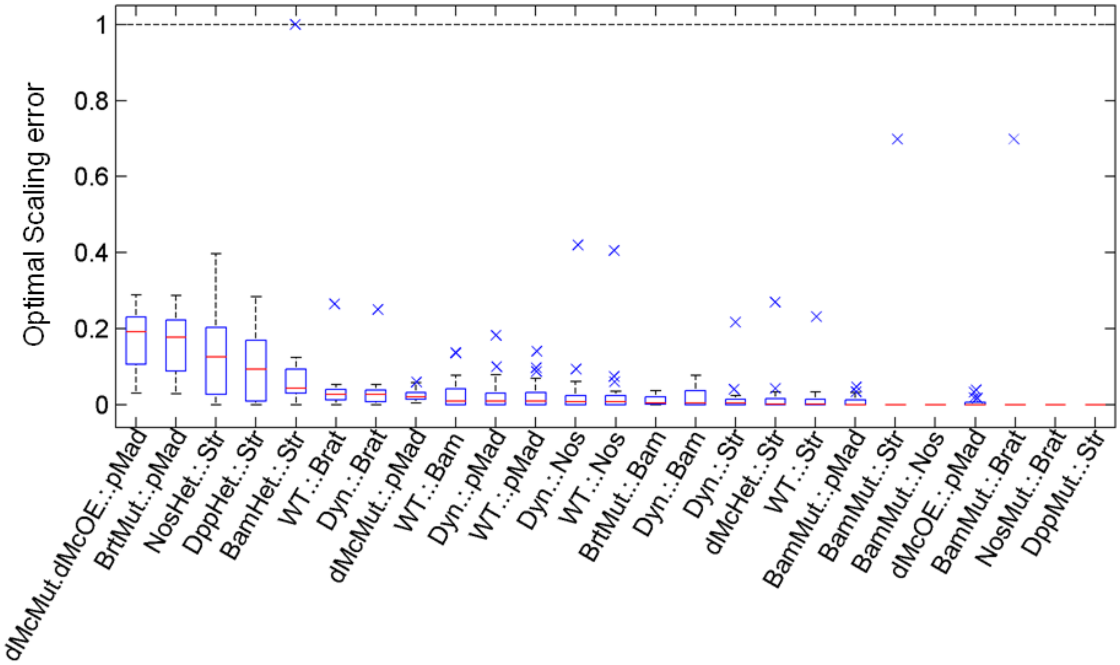


**Figure S6. Distribution of model error over the Piwi model.**

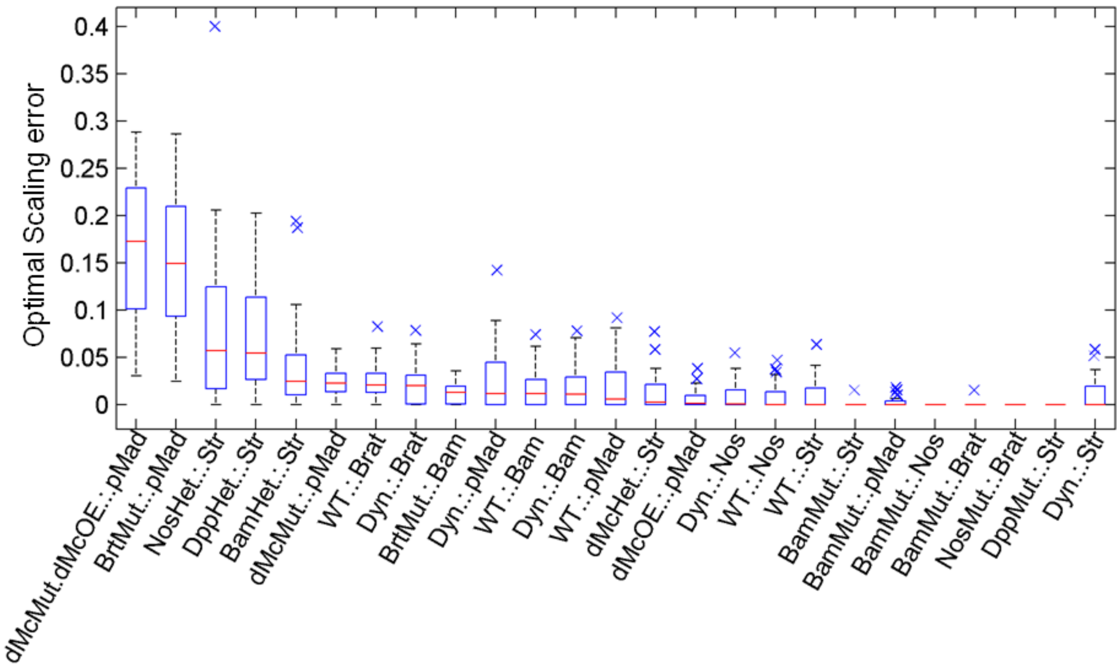


**Figure S7. Distribution of model error over the Argonaut model.**

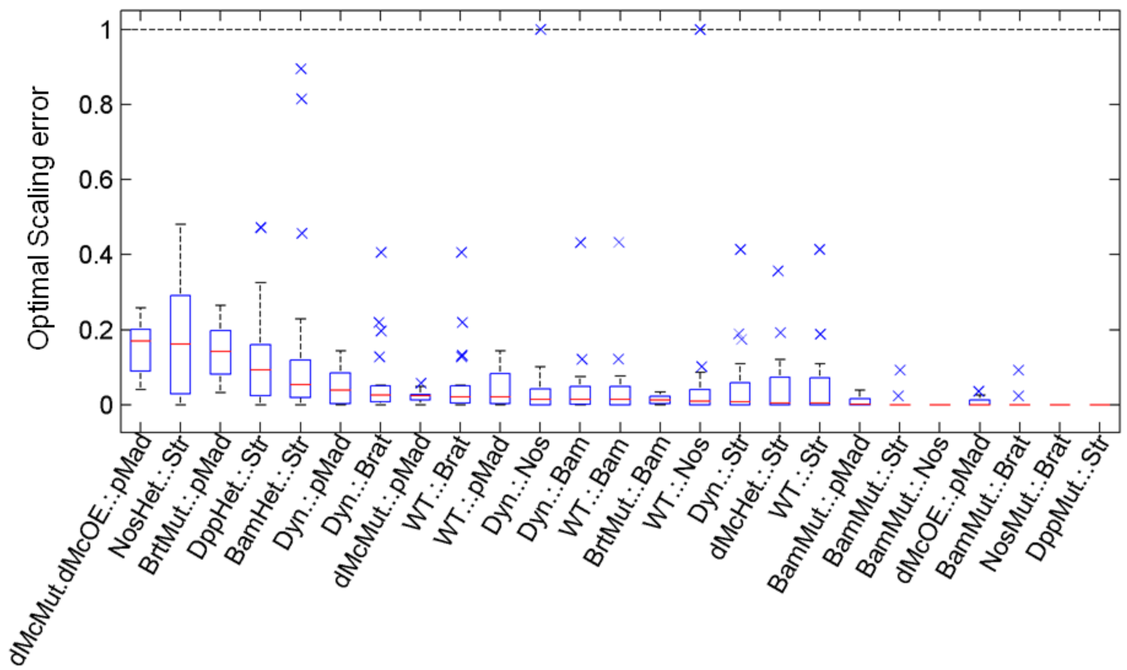


**Figure S8. Distribution of model error over the Diffusion model.**

## Sensitivity distribution

The distributions of the sensitivity of model outputs to parameters are provided here, for each model. The distributions shown include sensitivity of all outputs, over all Representatives, to each parameter (the derivative dY/dP, for states Y and parameters P). These distributions were examined to infer which parameters have little influence, and which are likely involved in the trade-offs across the Pareto front. The sensitivity distributions are plotted in order of the upper quartile value ($75^{th}$ percentile). Parameter names are predominantly represented as coded, and may not perfectly match the syntax used in the equations presented here. A dashed line at the top of the plot indicates truncation for visibility; any point lying on this line is valued greater than or equal to the value shown.

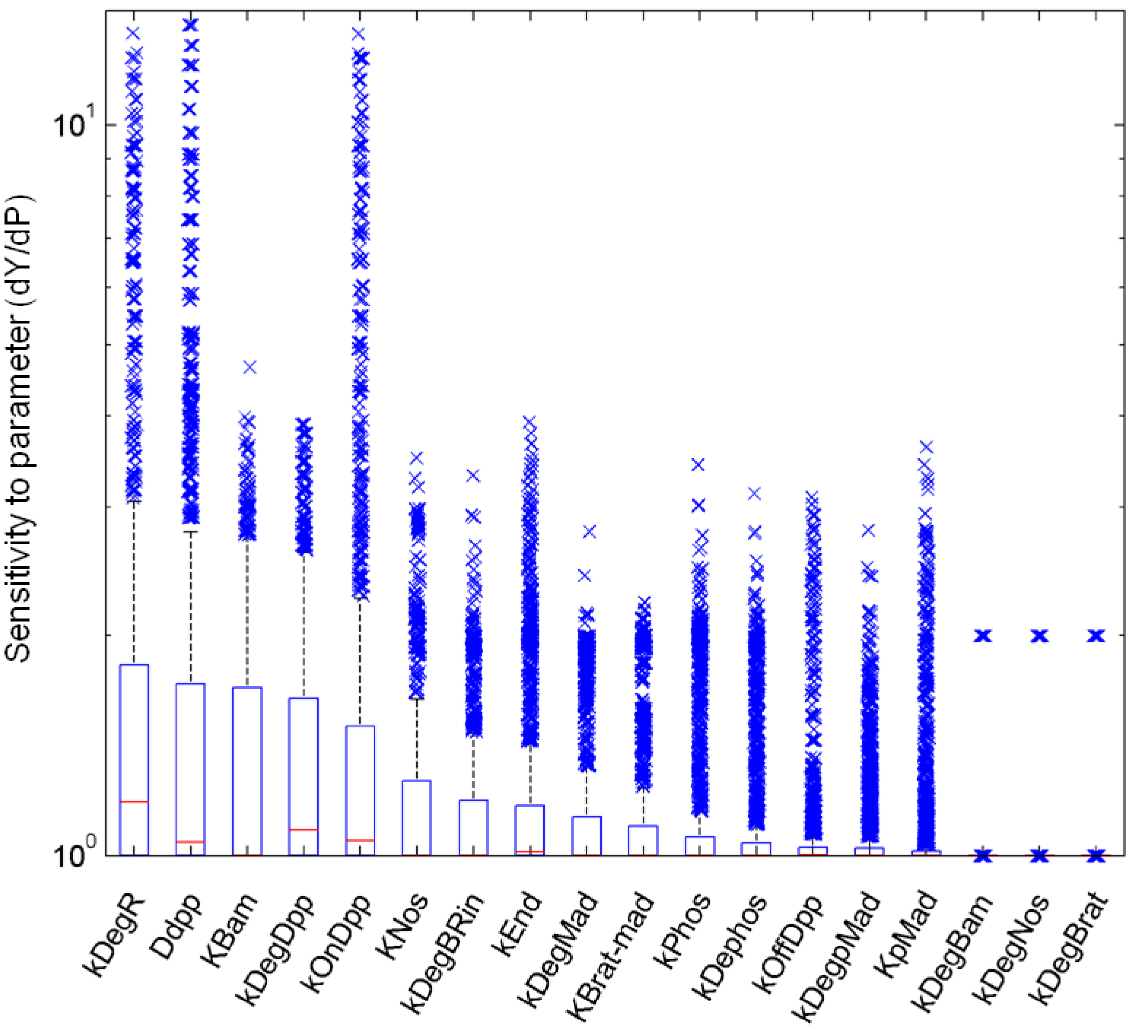


**Figure S9. Distribution of model sensitivity over the Core model.**

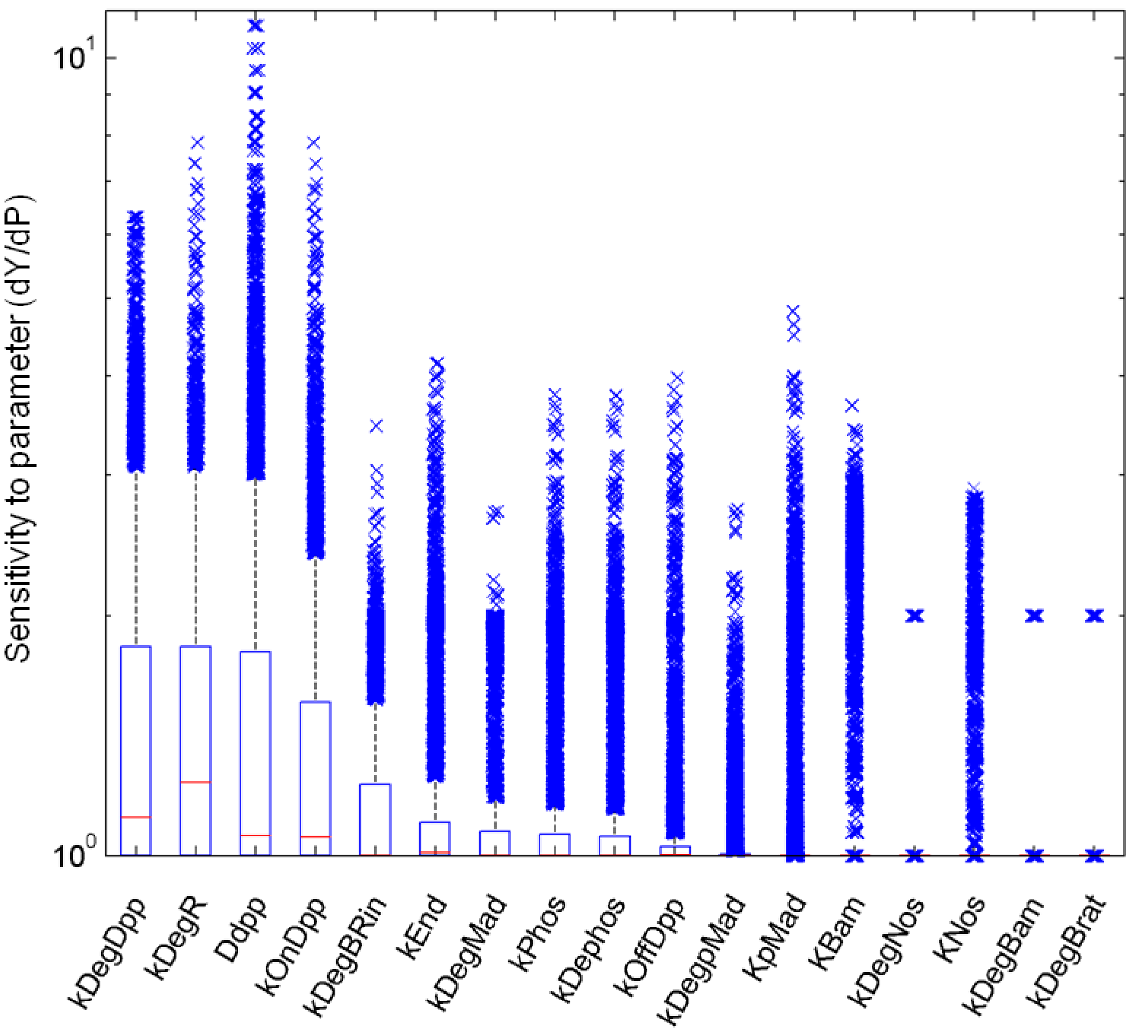


**Figure S10. Distribution of model sensitivity over the Alt1 model.**

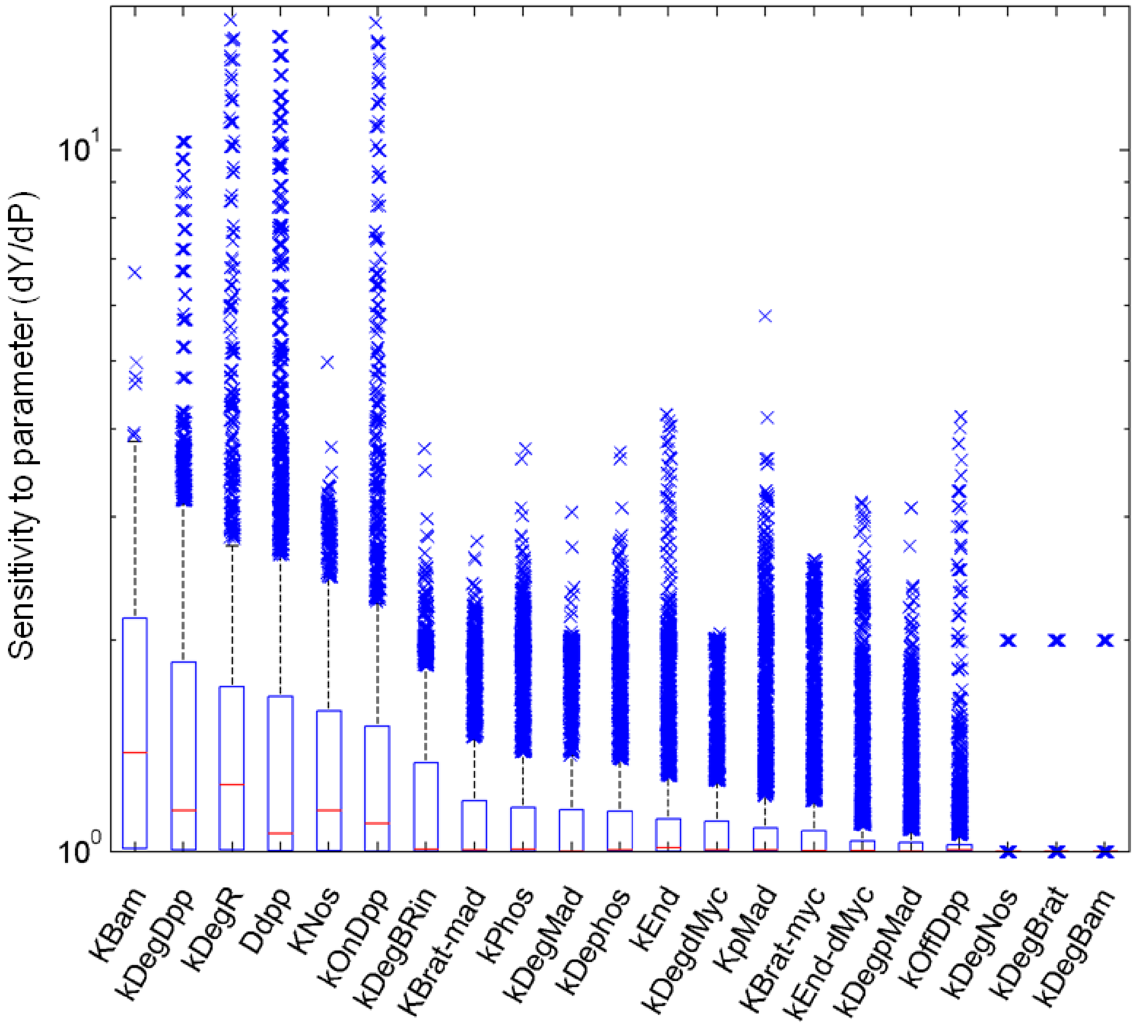


**Figure S11. Distribution of model sensitivity over the Endocytosis model.**

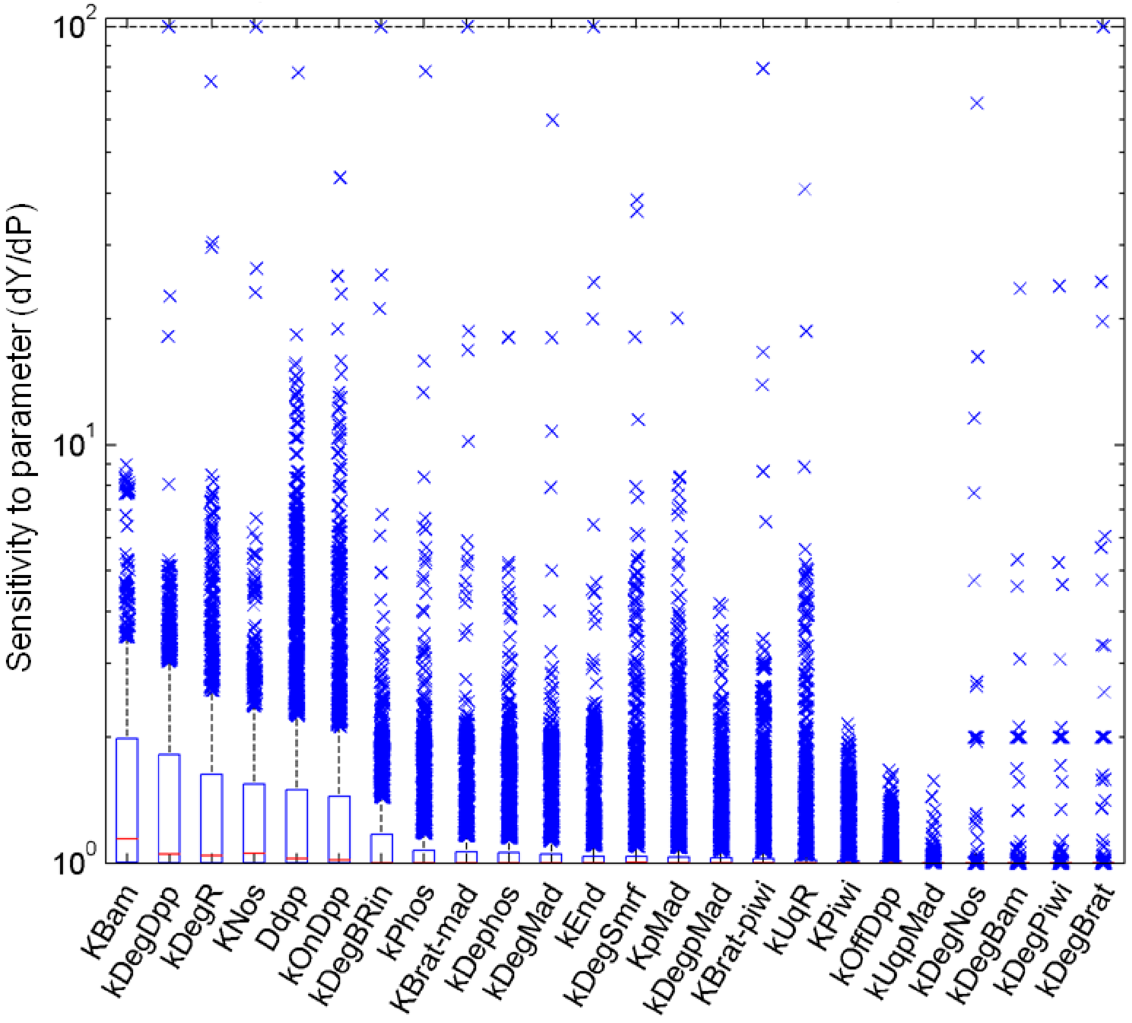


**Figure S12. Distribution of model sensitivity over the Piwi model.**

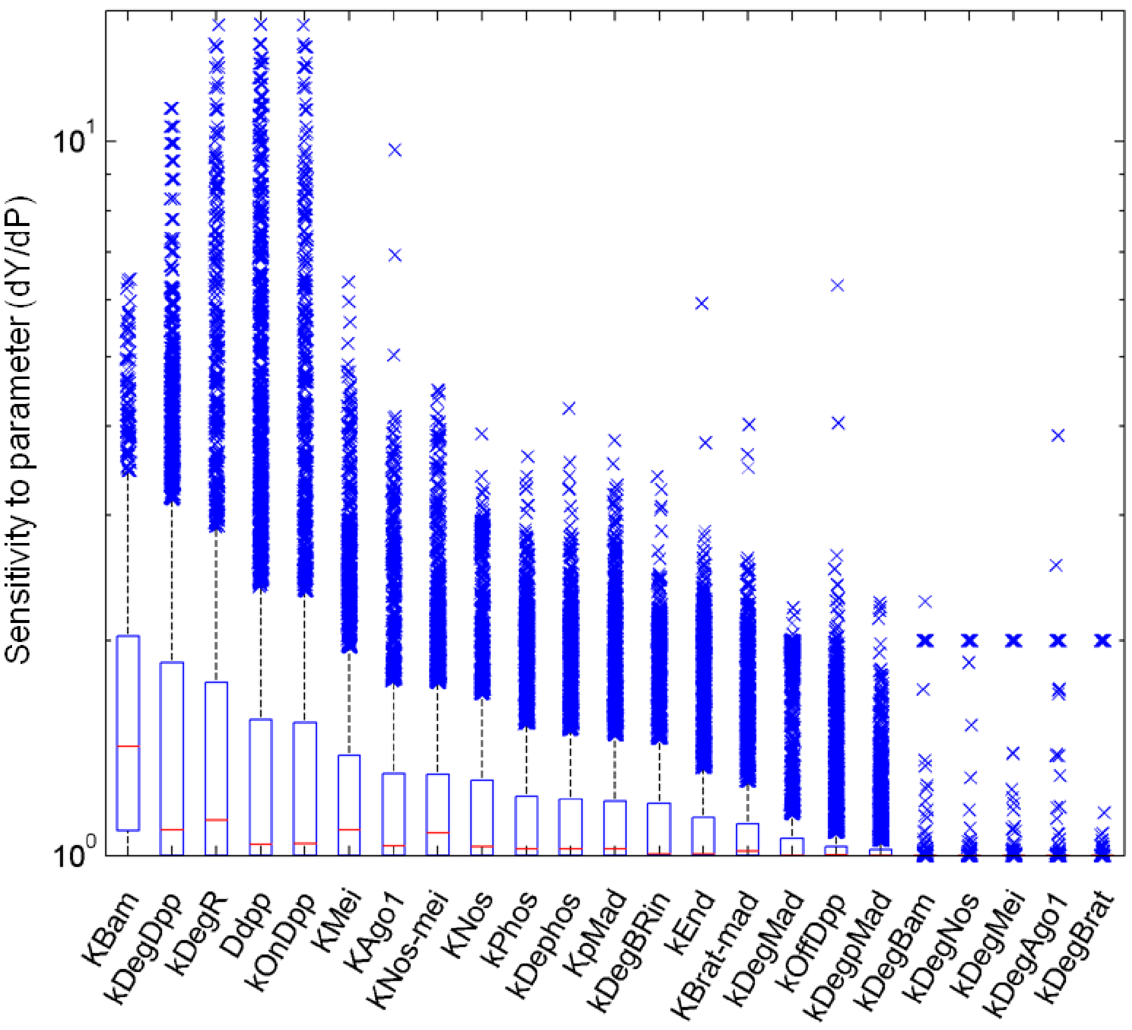


**Figure S13. Distribution of model sensitivity over the Argonaut model.**

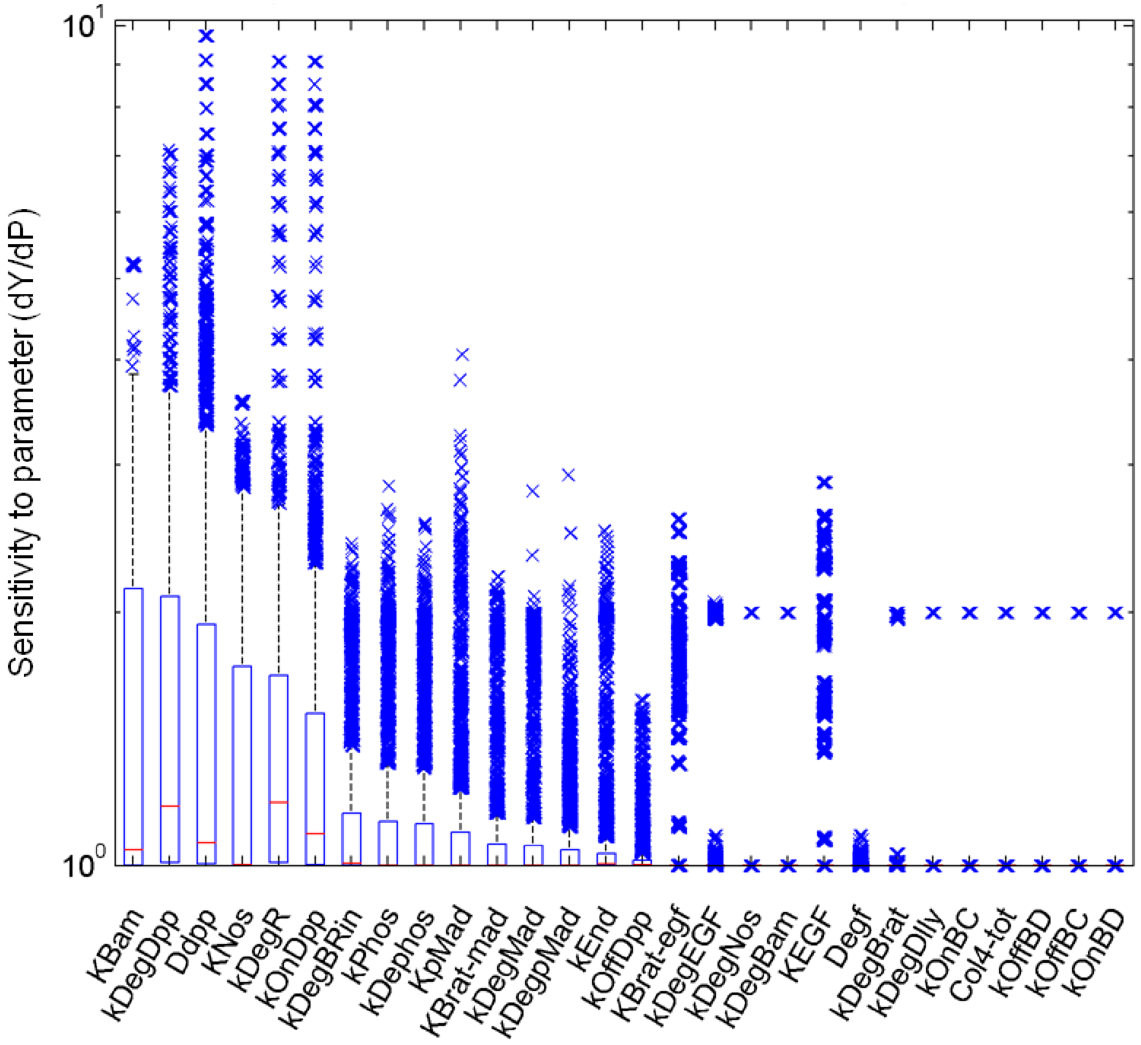


**Figure S14. Distribution of model sensitivity over the Diffusion model.**

## Experiment design

### Objective landscapes

The objective values for the best measurement for each of the experimental perturbations considered for qualitative data are shown in Figures S15-S17. Each objective landscape is sorted, with the abscissa showing the experiment number as sorted. The ordinate is the objective value, and in all cases higher values indicate more informative experiments.

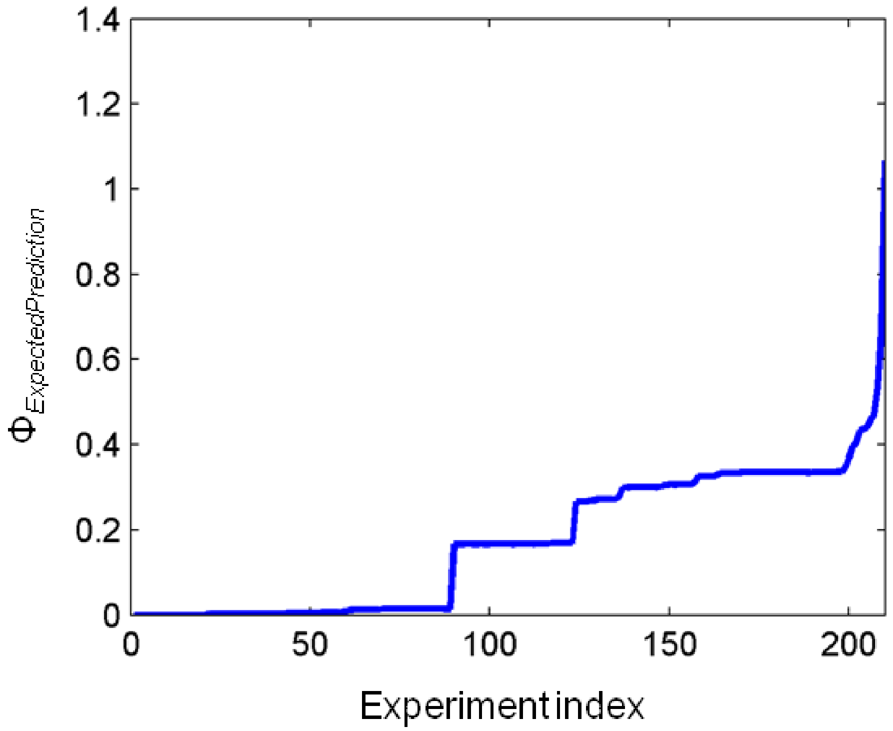


**Figure S15. Experiment design objectives by mean prediction.**

### Additional simulated experiments

Simulated experiments are provided in Figure S18 for the experiments that ranked highly in the design for model discrimination, but produced ‘flat’ outputs. Also included is an example of predictions from the design for quantitative data, by *mean sensitivity*.

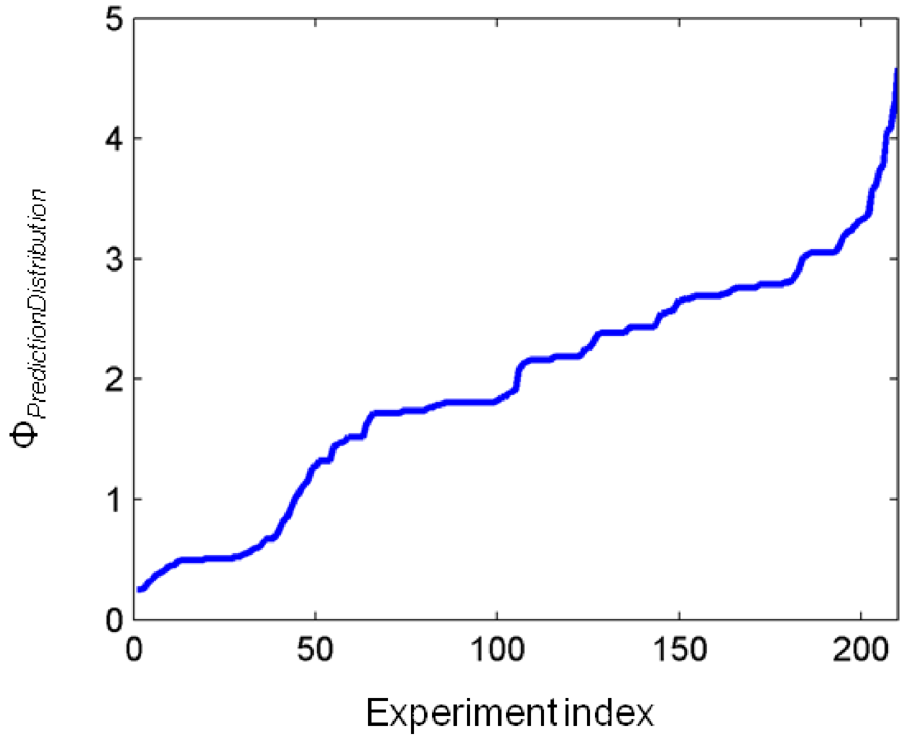


**Figure S16. Experiment design objectives by prediction distribution.**

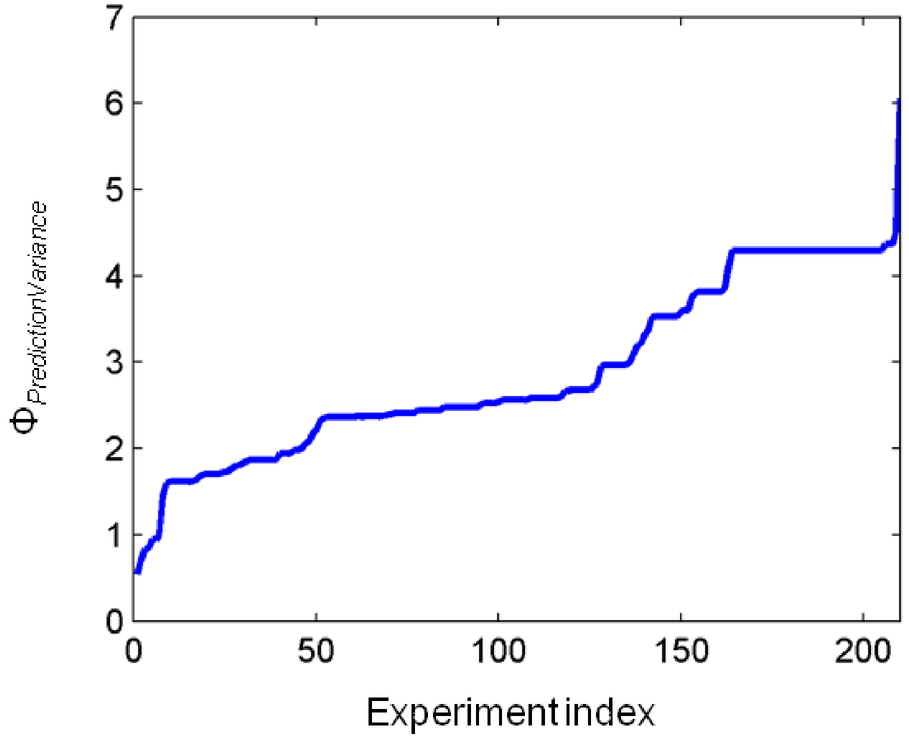


**Figure S17. Experiment design objectives to refine parameters.**

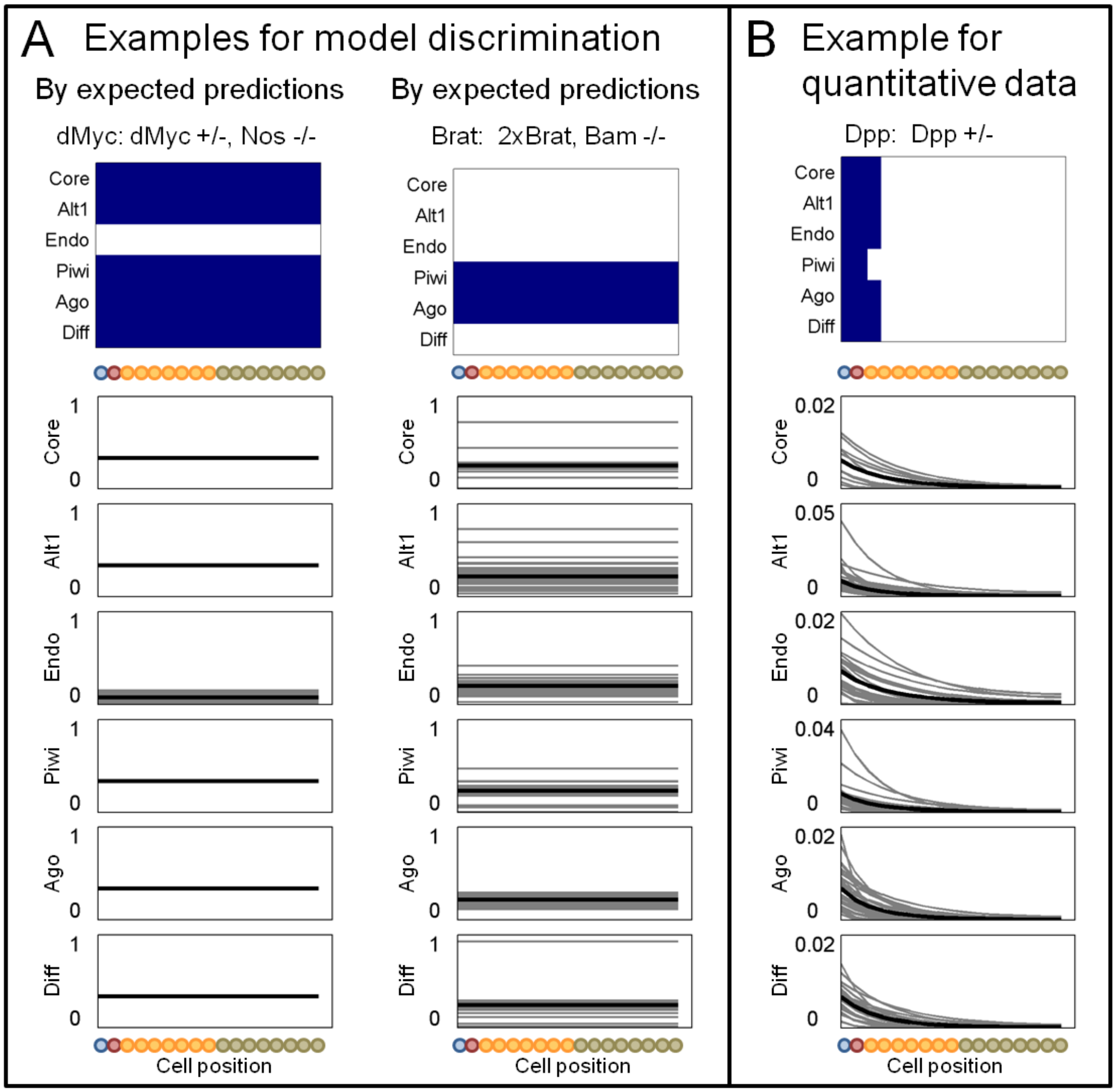


**Figure S18. Additional simulated experiments.** A) Highly ranked examples that predict 'flat' outputs across the germarium. B) Example predictions for quantitative data. Note the variance in absolute values (lower plots), despite limited variance in qualitative predictions (upper heatmap).
